# A human judgment approach to epidemiological forecasting

**DOI:** 10.1371/journal.pcbi.1005248

**Published:** 2017-03-10

**Authors:** David C. Farrow, Logan C. Brooks, Sangwon Hyun, Ryan J. Tibshirani, Donald S. Burke, Roni Rosenfeld

**Affiliations:** 1 School of Computer Science, Carnegie Mellon University, Pittsburgh, Pennsylvania, United States of America; 2 Department of Statistics, Carnegie Mellon University, Pittsburgh, Pennsylvania, United States of America; 3 Graduate School of Public Health, University of Pittsburgh, Pittsburgh, Pennsylvania, United States of America; CNRS, FRANCE

## Abstract

Infectious diseases impose considerable burden on society, despite significant advances in technology and medicine over the past century. Advanced warning can be helpful in mitigating and preparing for an impending or ongoing epidemic. Historically, such a capability has lagged for many reasons, including in particular the uncertainty in the current state of the system and in the understanding of the processes that drive epidemic trajectories. Presently we have access to data, models, and computational resources that enable the development of epidemiological forecasting systems. Indeed, several recent challenges hosted by the U.S. government have fostered an open and collaborative environment for the development of these technologies. The primary focus of these challenges has been to develop statistical and computational methods for epidemiological forecasting, but here we consider a serious alternative based on collective human judgment. We created the web-based “Epicast” forecasting system which collects and aggregates epidemic predictions made in real-time by human participants, and with these forecasts we ask two questions: how accurate is human judgment, and how do these forecasts compare to their more computational, data-driven alternatives? To address the former, we assess by a variety of metrics how accurately humans are able to predict influenza and chikungunya trajectories. As for the latter, we show that real-time, combined human predictions of the 2014–2015 and 2015–2016 U.S. flu seasons are often more accurate than the same predictions made by several statistical systems, especially for short-term targets. We conclude that there is valuable predictive power in collective human judgment, and we discuss the benefits and drawbacks of this approach.

## Author summary

Despite advanced and widely accessible health care, a large number of annual deaths in the United States are attributable to infectious diseases like influenza. Many of these cases could be easily prevented if sufficiently advanced warning was available. This is the main goal of epidemiological forecasting, a relatively new field that attempts to predict when and where disease outbreaks will occur. In response to growing interest in this endeavor, many forecasting frameworks have been developed for a variety of diseases. We ask whether an approach based on collective human judgment can be used to produce reasonable forecasts and how such forecasts compare with forecasts produced by purely data-driven systems. To answer this, we collected simple predictions in real-time from a set of expert and non-expert volunteers during the 2014–2015 and 2015–2016 U.S. flu seasons and during the 2014–2015 chikungunya invasion of Central America, and we report several measures of accuracy based on these predictions. By comparing these predictions with published forecasts of data-driven methods, we build an intuition for the difficulty of the task and learn that there is real value in collective human judgment.

## Introduction

### Context

It is perhaps unsurprising that societal advances in technology, education, and medicine are concomitant with reduced morbidity and mortality associated with infectious diseases [1]. This is exemplified by the staggering drop in infectious disease mortality in the United States during the 20th century [2]. Yet despite continued technological advances, the U.S. death rate due to infectious disease has not improved significantly since around the 1960s, and in fact has been rising since the 1980s [3]. It is evident that incremental advances in medical treatment are failing to reduce overall infectious disease mortality. One way in which the situation can be improved is through expanding our capacity for preparedness and prevention—we need forewarning [4]. This is the defining problem out of which the nascent field of epidemiological forecasting has risen.

There is widespread interest in predicting disease outbreaks to minimize losses which would otherwise have been preventable given prior warning. In recent years, the U.S. Centers for Disease Control and Prevention (CDC) has sponsored three challenges to predict Influenza epidemics [5–7], Defense Advanced Research Projects Agency (DARPA) has sponsored a challenge to predict the invasion of chikungunya [8, 9], several agencies working together under the Pandemic Prediction and Forecasting Science and Technology (PPFST) Working Group have sponsored a challenge to predict Dengue outbreaks [10], and the Research and Policy for Infectious Disease Dynamics (RAPIDD) group of the National Institutes of Health hosted a workshop for forecasting Ebola outbreaks [11]. Given the magnitude of time, energy, and resources collectively invested in these challenges by both participants and organizers, it is critical that qualitative and quantitative assessments be made to help understand where epidemiological forecasting excels and where it lags.

### Related work

As exemplified by the fields of meteorology and econometrics, statistical and computational models are frequently used to understand, describe, and forecast the evolution of complex dynamical systems [12, 13]. The situation in epidemiological forecasting is no different; data-driven forecasting frameworks have been developed for a variety of tasks [14–16]. To assess accuracy, forecasts are typically compared to pre-defined baselines and to other, often competing, forecasts. The focus has traditionally been on comparisons between data-driven methods, but there has been less work toward understanding the utility of alternative approaches, including those based on human judgment. In addition to developing and applying one such approach, we also provide an intuitive point of reference by contrasting the performance of data-driven and human judgment methods for epidemiological forecasting.

Methods based on collective judgment take advantage of the interesting observation that group judgment is generally superior to individual judgment—a phenomena commonly known as “The Wisdom of Crowds”. This was illustrated over a century ago when Francis Galton showed that a group of common people was collectively able to estimate the weight of an ox to within one percent of its actual weight [17]. Since then, collective judgment has been used to predict outcomes in a number of diverse settings, including for example finance, economics, politics, sports, and meteorology [18–20]. A more specific type of collective judgment arises when the participants (whether human or otherwise) are experts—a committee of experts. This approach is common in a variety of settings, for example in artificial intelligence and machine learning in the form of committee machines [21] and ensemble classifiers [22]. More relevant examples of incorporating human judgment in influenza research include prediction markets [23, 24] and other crowd-sourcing methods like Flu Near You [25, 26].

### Contribution

Here we assess the performance of a forecasting framework based on collective human judgment—“Epicast”. In particular, we assess its performance as a competitor in the aforementioned influenza and chikungunya forecasting challenges. Each of these challenges dealt with a different set of data and objectives, and we analyze them separately. For both influenza challenges, the data of interest was population-weighted percent influenza-like illness (wILI) in 10 regions of the U.S. and the U.S. as a whole. wILI is syndromic surveillance defined as the percent of patients having flu-like symptoms (fever over 100°F and either cough or sore throat) without a known cause other than influenza. wILI is reported voluntarily by health care providers through ILINet and distributed by CDC [27, 28]. The DARPA chikungunya challenge focused instead on predicting case counts within 55 countries and territories in the Americas. The data of interest was the number of weekly cases (including suspected, confirmed, and imported) within each location, cumulatively since the beginning of 2014. These case counts are published by the Pan American Health Organization (PAHO) [29].

In regards to terminology in subsequent discussion, there is an important distinction to be made between a *prediction* and a *forecast*. These words are often used interchangeably elsewhere, but here we use them to refer to subtly different concepts. A prediction makes an absolute statement about the future and says nothing about other potential outcomes. In contrast, a forecast is a generalization of a prediction in which a probability is assigned to all possible outcomes. In the case of Epicast, we collect a prediction from each participant—a single possibility. Epicast aggregates many such predictions to produce a forecast—a probability distribution over all possibilities. Because predictions and forecasts make different claims, they are evaluated by different metrics; we use mean absolute error (MAE) to assess predictions and mean negative log likelihood (based on “logarithmic score” [30], also known as “surprisal” in other contexts [31]) to produce a figure of merit for forecasts.

As part of our evaluation of the Epicast system in forecasting flu, we compare with several competing statistical and/or data-driven systems. For the 2014–2015 flu contest, we compare Epicast with “Empirical Bayes” and “Pinned Spline”; for the 2015–2016 flu contest, we compare Epicast with “Stat” and “ArcheFilter”. All of these systems were serious and successful competitors in their respective contest years. Consequently, the performance of these systems provides a measure of the state of the art in flu forecasting. None of these competing systems were used in both flu contests, hence we compare against a different set of systems in different years.

The Empirical Bayes system [32] is based on the notion that future epidemics will generally resemble past epidemics, up to basic transformations and noise. This system defines a prior distribution over wILI trajectories, draws samples from that distribution, assigns a likelihood-based weight to each sample, and finally produces a posterior distribution over trajectories. The reported forecast for each target is derived from the posterior distribution of wILI trajectories.

The Pinned Spline system [33] attempts to smoothly interpolate current and past wILI. To do this, two partial wILI trajectories are defined. The first partial trajectory spans the start of the season through the current week and is defined to be wILI as published by CDC for the current season. The second partial trajectory spans the next week through the end of the season and is defined to be the week-wise mean of the wILI trajectories of past epidemics over the same span. Finally, the two partial trajectories are connected with smoothing splines. In subsequent analysis we only evaluate point predictions—not forecasts—made by the Pinned Spline system.

The Stat system is a weighted ensemble of statistical methods, including both Empirical Bayes and Pinned Spline. It additionally contains baseline components (including a uniform distribution and an empirical distribution) and other non-mechanistic methods (including delta density and kernel density methods). The cross-validation weight assigned to each constituent method is recomputed for each forecast. Stat forecasts are a weighted combination of the forecasts of each method.

The ArcheFilter system [33] assumes that there is a latent archetype wILI trajectory, and that the observed wILI trajectory of each flu epidemic is a transformed, noisy version of the archetype. The ArcheFilter defines this archetype roughly as the peak-aligned, week-wise mean wILI trajectory of all past epidemic seasons. As an epidemic progresses, a Kalman filter is used to estimate the time-shift and wILI-scale parameters that, when applied to the archetype, most parsimoniously explain the observed wILI trajectory of the current epidemic. Uncertainty in the state of the filter—the shift and scale parameter values—gives rise to a distribution over wILI trajectories from which the forecast for each target is derived.

## Methods

### Ethics statement

This study was granted Carnegie Mellon University IRB exemption with ID STUDY2015_00000142.

### Collecting predictions

**Fig 1:**
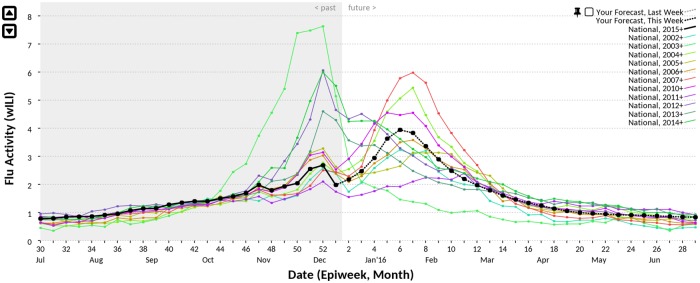
Epicast user interface. A screenshot of the Epicast user interface for predicting flu in the United States. On any given week, the wILI trajectory of the current season (solid black) is only partially observed. A user’s prediction is a continuation of this trajectory over weeks in the future (dashed black). wILI trajectories of past seasons show the typical course of influenza epidemics (colored lines).

We developed a website [34] for collecting predictions of epidemiological time series (Fig 1). Participants were shown a partial trajectory and were asked to hand-draw a continuation of the trajectory as a prediction. At regular intervals, user-submitted trajectories were collected and an aggregate forecast was generated. Participants were not shown the predictions made by other participants. We produced, in real-time, forecasts for the 2014–2015 and 2015–2016 U.S. flu seasons and predictions for the 2014–2015 chikungunya invasion of the Americas. For influenza, we collected predictions from the general public; for chikungunya, we only collected predictions from a set of selected experts in related fields. All three forecasting challenges were carried out as the event (epidemic or invasion) progressed; we did not produce retrospective forecasts.

Each week during the 2014–2015 and 2015–2016 flu seasons, we asked participants to predict wILI for each remaining week of the season. Each individually submitted prediction was a trajectory of varying length (depending on the week of submission) of wILI values, and we asked users to provide such predictions for each of the HHS and U.S. regions. Similarly, once each month, from August, 2014 through January, 2015, we asked participants to predict the cumulative weekly chikungunya case count in each of the 55 Pan American Health Organization (PAHO) locations through the end of February 2015.

Each predicted trajectory was extended to cover the entire time series of interest by concatenating the observed time series at the time of submission with the predicted time series. By doing this, all time series were made to have the same length (for example, 32 weekly wILI values spanning the 2014–2015 flu season).

### Forecasting targets

The objectives of the influenza challenges were to forecast a number of features, or “targets”, of the weekly wILI time series. These included: “Peak Height”, the maximum value of wILI reported throughout the flu season; “Peak Week”, the Morbidity and Mortality Weekly Report (MMWR) week number [35] on which wILI reaches its maximum value; and the next four values of wILI, called “1–4 Week Lookaheads”. Each of these values was forecasted separately for each of the ten Health and Human Services (HHS) regions of the U.S. and also for the U.S. as a whole—a super-region which we include in our analysis as one of eleven total regions. The forecast for each target in each region consisted of a set of probability bins over a pre-defined range of outcomes and a prediction of the single most likely outcome. The chikungunya challenge objective was simply to predict the trajectory of cumulative case counts in each country or territory.

Of the six flu targets, two are season-wide: Peak Week and Peak Height. The rest—the 1–4 Week Lookaheads—are short-term targets. Peak Week is an integer representing the number of weeks elapsed between the start of the season and the week during which wILI peaks. The start of the season is defined as MMWR week 40 of the first year of the season, written as “2014w40” or “2015w40”, depending on the season. The remaining five targets are measured in wILI on a continuous scale from 0% to 100%. An additional season-wide target, the MMWR week of epidemic onset, is discussed in S1 Text.

### Aggregating predictions

The Epicast point prediction for any target was defined as the median of the target values measured on user predictions. The Epicast forecast for any target was a Student’s t distribution with location equal to the median value (the point prediction), scale equal to the sample standard deviation of values, and degrees of freedom equal to the number of participants. The same general methodology was used for both forecasting challenges, with the exception that we produced both a prediction and a forecast for influenza and only a prediction for chikungunya.

### Evaluating outcomes

We primarily assess the quality of predictions in terms of mean absolute error (MAE). Given a set of *N* true outcomes, *y*, and corresponding predictions, $\hat{y}$, MAE can be written as:

$$\text{MAE}=\frac{1}{N}\sum_{i=1}^{N}|y_{i}-{\hat{y}}_{i}|\text{.}$$

We further assess wILI and case-count predictions (for example, Peak Height) by measuring how often predictions fall within some range (± 10%, 20%, 30%, 40%, or 50%) of ground truth. We similarly assess predictions of weeks (for example, Peak Week) by measuring how often predictions fall within some range (± 1, 2, 3, 4, or 5 weeks) of ground truth. We report the fraction of the time that predicted values fall within each range, aggregated over regions and potentially also weeks. We refer to these analyses as “fraction of predictions accurate within a target range”.

We assess the quality of flu forecasts in terms of a likelihood-based score. We define the “log score” as the negative average of the logarithm of the probability assigned to a range of values surrounding the true outcome. In the case of Peak Week, we consider the log score of the range of the actual Peak Week plus or minus one week (for example, if the Peak Week was 5, we compute the log likelihood of the probability assigned to a peak being on week 4, 5, or 6). Suppose that ${\text{PkWk}}_{r}^{\text{obs}}$ denotes the observed value of Peak Week in region *r* and that P(⋯) represents the probability assigned by the forecaster to a given outcome. Then the score across all regions can be written as:

$$\text{score}=-\frac{1}{11}\sum_{r=1}^{11}\log\text{P}\left({\text{PkWk}}_{r}\in \left[{\text{PkWk}}_{r}^{\text{obs}}-1,{\text{PkWk}}_{r}^{\text{obs}}+1\right]\right).$$

For the five wILI targets, we only have available a set of probability bins each of width 1 wILI, as this is what was required by CDC’s 2014–2015 flu contest. To determine which bins to include in the likelihood calculation, we select (1) the wILI bin containing the actual value and (2) the adjacent wILI bin nearest to the actual value. For example, the actual Peak Height in the U.S. National region was 6.002, and we select the two bins which together give the probability assigned to the event that actual Peak Height falls between 5 and 7. Suppose a forecast was made that P(5 ≤ wILI < 6) = 0.215 and P(6 ≤ wILI < 7) = 0.412; the log score assigned to this forecast is − log(0.215 + 0.412) = 0.467. For Peak Height (and similarly for the Lookahead targets) across all regions:

$$\text{score}=-\frac{1}{11}\sum_{r=1}^{11}\log\text{P}\left({\text{PkHt}}_{r}\in \left[\text{round}\left({\text{PkHt}}_{r}^{\text{obs}}\right)-1,\text{round}\left({\text{PkHt}}_{r}^{\text{obs}}\right)+1\right]\right).$$

To compensate for the varying difficulty over time of predicting and forecasting, we often treat accuracy as a function of “lead time”, the number of weeks preceding the region-specific Peak Week. Positive, zero, and negative lead times indicate predictions and forecasts made before, on, and after the epidemic peak, respectively. We consider lead times for the 2014–2015 flu season that range from +10 to −10 weeks. However, due to the unusually late Peak Week within most regions in the 2015–2016 flu season, we constrain lead times in that season to the range of +10 to −5 weeks.

To contextualize the accuracy of both predictions and forecasts, we compare Epicast with individual participants and/or other forecasting methods. To further contextualize log scores, we show also the log score of a hypothetical “Uniform” system in which uniform probability is assigned to all plausible outcomes. For Peak Week, we define this as a uniform distribution over weeks 2014w46 through 2015w12 and 2015w45 through 2016w12 ($p=\frac{1}{20}$ per week, per season), and for the wILI targets we define this as a uniform distribution over 0% to 12% wILI ($p=\frac{1}{12}$ per bin). The Uniform system is intended to provide a lower bound on the performance of a reasonable forecaster.

Our main challenge in presenting results is that the space in which comparisons can be made consists of several orthogonal dimensions: regions (U.S. and 10 HHS regions), targets (Peak Week, Peak Height, and 1–4 Week Lookaheads), season weeks (depending on season and target, up to 32), and error metrics (MAE and log score). To concisely compare system performance, we are given the non-trivial task of reducing this dimensionality, otherwise we would come to thousands of separate figures of merit. Several confounding issues impede aggregation along any one axis; forecasting difficulty varies over time as the season progresses, the various regions may peak at different times in the season, long-term targets are often more difficult to predict than short-term targets, and targets are measured in different units. To work around these complications in the case of point predictions, we rank systems and participants in terms of absolute error and perform our analysis on the relative ranking assigned to each predictor. More specifically, we consider the pairwise ranking in absolute error of Epicast versus individual participants and statistical frameworks. For each lead time, region, and target, we ask whether Epicast or the competitor had a smaller absolute error, and we measure the fraction of instances where Epicast had the smaller error—a “Win Rate”. To assess the statistical significance of each result, we use a Sign test with the null hypothesis that the pair of forecasters is equally accurate. It should be noted that this test assumes that all observations are independent, but results across adjacent weeks, for example, are likely to be correlated to some extent.

We define ground truth to be the version of wILI published by CDC 15 weeks after the end of each flu season—MMWR week 35. Specifically, we use values published on 2015w35 and 2016w35 for evaluating the results of the 2014–2015 and 2015–2016 flu contests, respectively.

## Results

### Influenza

**Fig 2:**
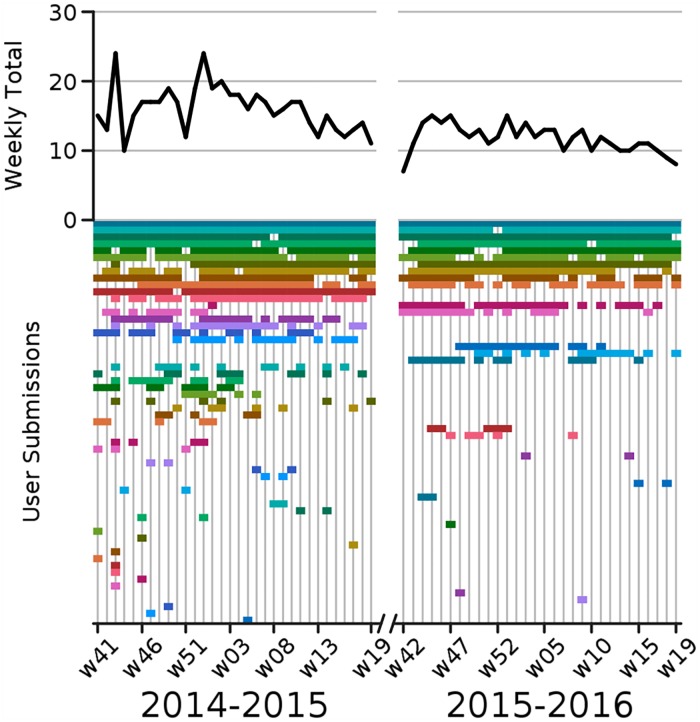
Overview of Epicast participation. **Top:** time series of the number of active participants per week. **Bottom:** timeline of weekly participation. (Assigned colors are for visualization purposes only and are consistent across figures.).

For the 2014–2015 flu season we gathered a total of 5,487 trajectories from a set of 48 volunteer participants during the 32 week period spanning 2014w41 through 2015w19. For the 2015–2016 flu season we gathered a total of 3,833 trajectories from a set of 23 volunteer participants during the 30 week period spanning 2015w42 through 2016w19. Participants varied in self-identified skill, from experts in public health, epidemiology, and/or statistics, to laypersons. Participation varied over time with an average of 16 participants per week during the 2014–2015 season and 12 participants per week during the 2015–2016 season (Fig 2). In the following analysis we did not handle expert and non-expert predictions differently, but we compare the performance of the two groups in S1 Text—the experts on average made slightly more accurate predictions. In what follows, we group errors across regions for brevity, but a breakdown of performance within each region is also given in S1 Text.

**Fig 3:**
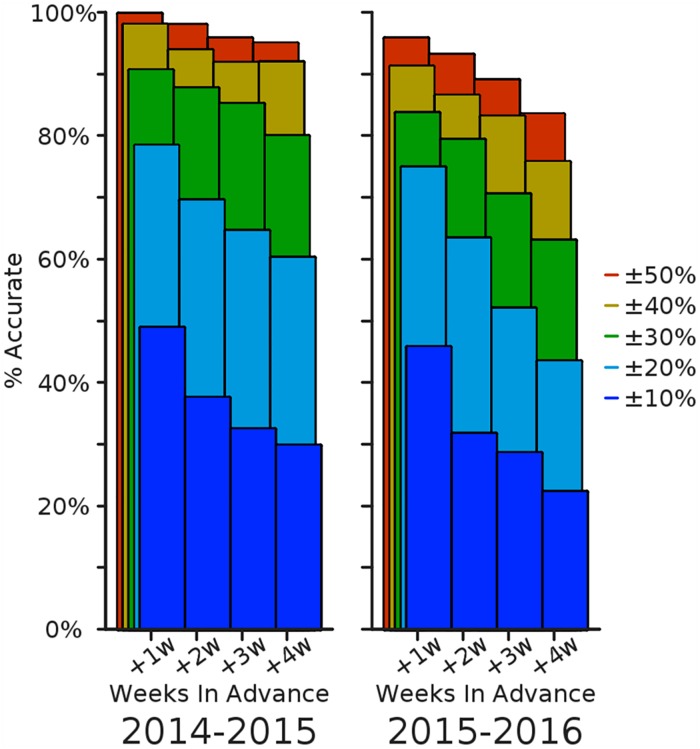
Relative prediction accuracy on short-term targets. The percent of regions and submission weeks (*n*_2014_ = 352, *n*_2015_ = 330) where the Epicast point prediction was accurate within some range of the actual value is shown separately for each of the four short-term targets.

We first consider the fraction of predictions accurate within a target range, aggregated over weeks of the season (Fig 3). For the four short-term Lookahead targets, the Epicast prediction is within 10% of the actual value just under half the time when predicting one week into the future; this falls to roughly one quarter of the time when predicting 4 weeks into the future. The trend is similar, though perhaps less abrupt, at other accuracy thresholds. Accuracy within 50% is achieved near or above 85% of the time, even predicting up to 4 weeks ahead.

**Fig 4:**
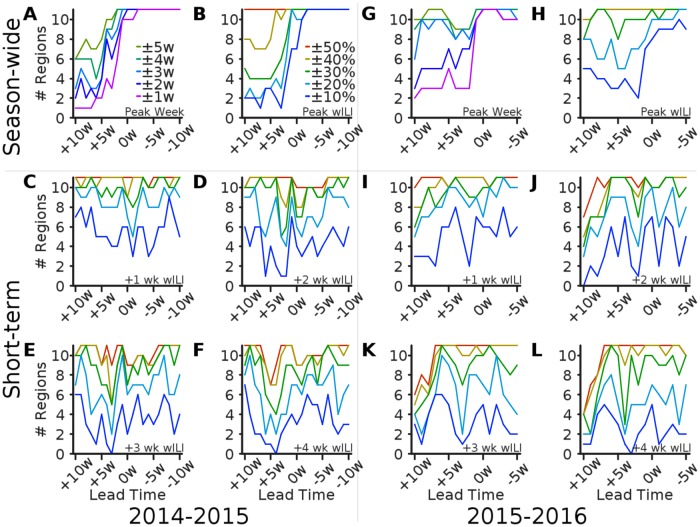
Number of regions accurate within a target range by lead time. The number of regions where the Epicast point prediction was accurate within some range of the actual value is plotted as a function of lead time. Subplots show accuracy in **(A; G)** Peak Week, **(B; H)** Peak Height, and **(C–F; I–L)** wILI at 1, 2, 3, and 4 weeks ahead, respectively.

We next consider the number of regional predictions accurate within a target range, as a function of lead time (Fig 4). For 2, 3, and 4 weeks ahead, the lead time with lowest accuracy is roughly 2, 3, and 4 weeks ahead of the Peak Week, respectively, which suggests that there is a distinct challenge in forecasting the Peak Height. This is to be expected because there is significantly more volatility around the peak of the epidemic. In general, accuracy in season-wide targets rises sharply 2–5 weeks before the epidemic peak and remains high through the remainder of the season.

For the 2015–2016 season, short-term accuracy (relative, not absolute) is surprisingly low in several regions around 10 weeks before the peak. This is likely due to the fact that the Peak Week in most regions was exceptionally late during this season. As a result, wILI was still near to baseline values for several weeks after predictions were made. Predicting a premature rise in wILI when ground truth was small in magnitude resulted in errors exceeding 50% of the actual value in several regions. Once it became clear that this would likely turn out to be a mild and/or late-peaking season, accuracy rose to nominal pre-peak levels.

**Fig 5:**
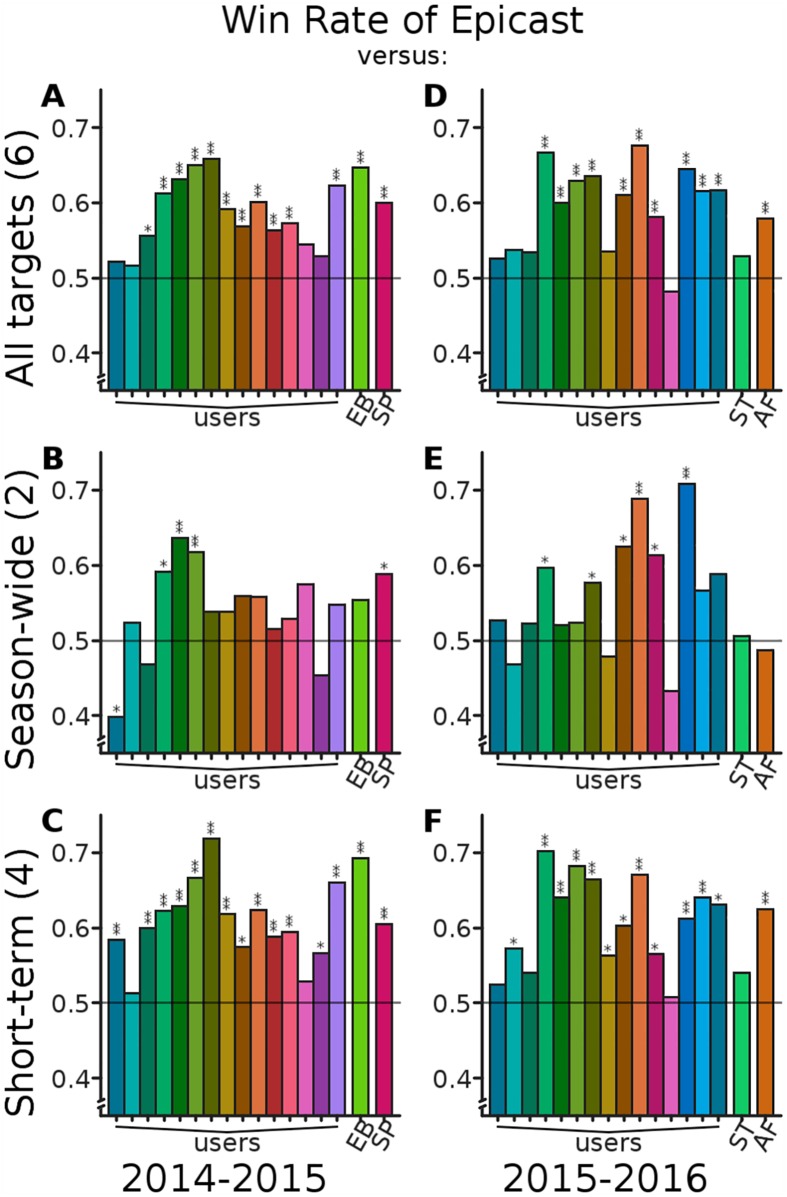
Epicast Win Rate against individuals and competing systems. All plots show, for each predictor (users participating on at least half of the weeks and statistical systems Empirical Bayes (EB), Pinned Spline (SP), Stat (ST), and ArcheFilter (AF)), Win Rate: the fraction of instances where Epicast had lower absolute error than the competitor, across all regions and lead times (*n*_2014_ = 231, *n*_2015_ = 176 per target). A Win Rate above the reference line of 0.5 implies that Epicast had lower absolute error more frequently than the indicated predictor. Statistical significance is determined by Sign test; *: *p* < 10^−2^; **: *p* < 10^−5^. Subplots show Win Rate considering **(A; D)** all six targets, **(B; E)** the two season-wide targets, and **(C; F)** the four short-term targets.

The remainder of our analysis is focused on comparing the accuracy of Epicast with individual participants and competing methods; we begin with Win Rate (Fig 5). Overall, considering all targets, Epicast has Win Rates above 0.5 (lower absolute error than a competitor on a majority of predictions) when compared with all but one individual participant and all four statistical frameworks. In season-wide targets, Epicast performs reasonably well; however, six participants and the ArcheFilter method bring Epicast’s Win Rate below 0.5. In short-term targets, Epicast has Win Rates uniformly above 0.5. Epicast has a Win Rate significantly higher than the Spline method in all categories and significantly higher than the Empirical Bayes and ArcheFilter methods both overall and in short-term targets. Epicast never has a significantly lower Win Rate than any of the competing statistical systems.

**Fig 6:**
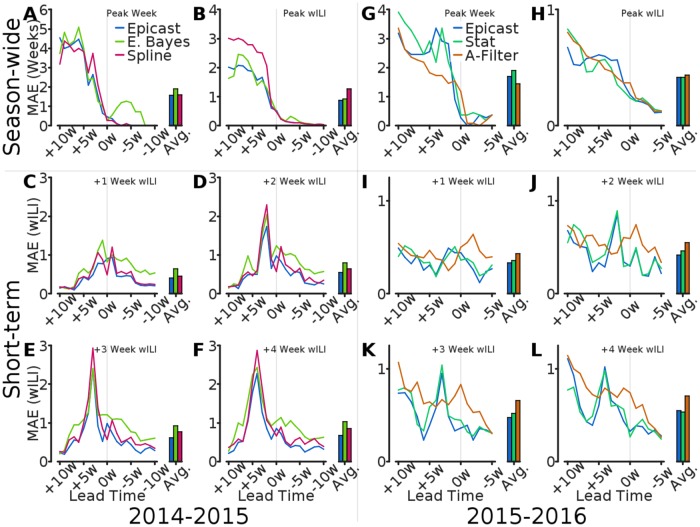
Comparison of mean absolute error by lead time. Mean absolute error across regions (*n* = 11) is plotted as a function of lead time for of Epicast, Empirical Bayes (E. Bayes), Pinned Spline (Spline), Stat, and ArcheFilter (A-Filter). MAE averaged over lead times is shown for each method on the right side of each subplot. Subplots show MAE in **(A; G)** Peak Week, **(B; H)** Peak Height, and **(C–F; I–L)** wILI at 1, 2, 3, and 4 weeks ahead, respectively.

Next, we compare predictions in terms of MAE. We calculate, separately for each target, MAE across regions as a function of lead time (Fig 6). In agreement with previous results, Epicast MAE in season-wide targets generally decreases with lead time and is highest in short-term targets when predicting the peak value of wILI. MAE is occasionally elevated in short-term targets on the Peak Week (lead time = 0), suggesting a relative increase in uncertainty immediately after the true peak (which is not known at the time to be so). Compared with the statistical methods, Epicast particularly excels when predicting short-term targets.

**Fig 7:**
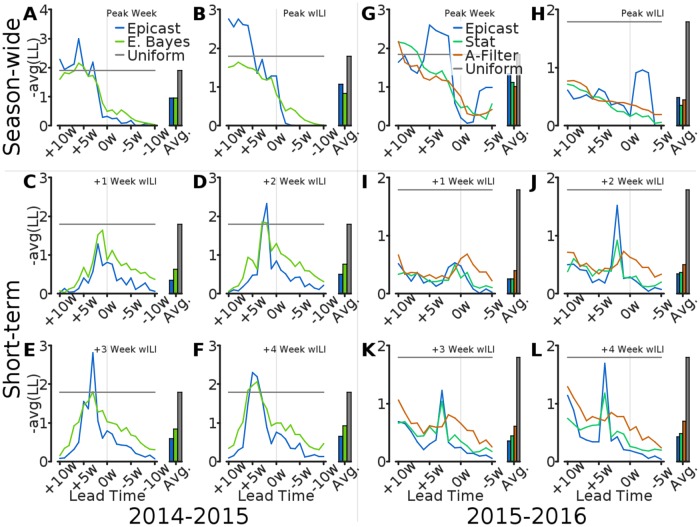
Comparison of log score by lead time. Log score, averaged across regions (*n* = 11), is plotted as a function of lead time for of Epicast, Empirical Bayes (E. Bayes), Stat, ArcheFilter (A-Filter), and Uniform. Log score averaged over lead times is shown for each method on the right side of each subplot. Subplots show log score by **(A; G)** Peak Week, **(B; H)** Peak Height, and **(C–F; I–L)** wILI at 1, 2, 3, and 4 weeks ahead, respectively.

Finally, we compare forecasts in terms of log score. Our analysis in this context does not include the 2014–2015 Spline method, but the hypothetical Uniform method is included for both seasons. We compute the average log score for Epicast and competing methods separately for each target and lead time (Fig 7). In the 2014–2015 season, Empirical Bayes scored within the bounds of the Uniform system more consistently than Epicast. However, in the 2015–2016 season, all systems consistently scored within the Uniform bounds for wILI targets, likely due at least partly to a relatively low peak wILI in this season. Across both seasons, Epicast has average log score in short-term targets as good as, or better than, that of the statistical systems. However, the statistical systems almost uniformly outperform Epicast in season-wide targets.

### Chikungunya

**Fig 8:**
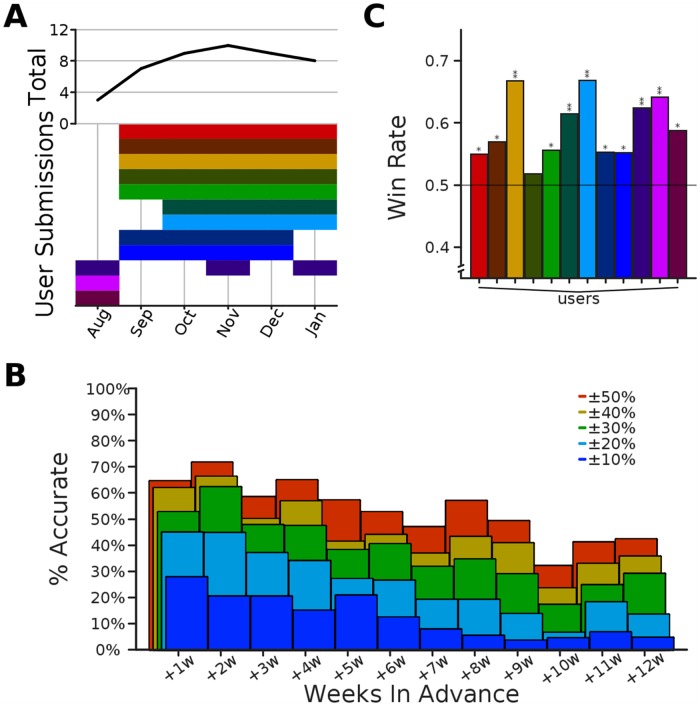
Overview of chikungunya predictions. **(A)** Similar to Fig 2, participation is shown per month (top) and per expert (bottom). **(B)** As in Fig 3, percent of predictions within some range of the target value as a function of the number of weeks in advance that the prediction was made (45 ≤ *n* ≤ 84). **(C)** As in Fig 5, the fraction of instances where Epicast had lower absolute error than each individual participant, across all countries and weeks (336 ≤ *n* ≤ 795; Sign test; *: *p* < 10^−2^; **: *p* < 10^−5^).

In total, we gathered 2,530 trajectories from a set of 12 volunteers with expertise in vector-borne diseases, public health, and/or epidemiology (Fig 8). Predicting chikungunya fundamentally differed in two ways from predicting flu. First, the chikungunya invasion of the Americas was a rare event for which little historical precedent was available, whereas flu epidemics are a regular occurrence for which we have significant historical data. Second, errors in (cumulative) chikungunya predictions accumulated over weeks, whereas errors in (non-cumulative) flu predictions were separated out across weeks. While it would have been trivial to convert a cumulative trajectory into a non-cumulative trajectory, the published counts which were defined to be ground truth are only available sporadically over time, preventing us from converting the true cumulative trajectory into a non-cumulative trajectory. The increased difficulty of the task is reflected by a reduction in accuracy. At best (1 week ahead), less than one in three predictions were within 10% of the actual value; and at worst (10 weeks ahead), over half of the predictions were off target by more than 50%. Even in such conditions, when comparing pair-wise absolute error between Epicast and each user, Epicast more frequently predicts closer to the true value than any individual user.

## Discussion

### Outcomes

Epicast was one of two winning methods in the 2014–2015 flu contest [7]. Epicast was one of three winning methods in the 2015–2016 flu contest [36] (the other winners were Stat and ArcheFilter). We expect a future CDC publication to provide additional details and analysis. Epicast was not selected as one of the six chikungunya challenge winners [37], but we are told that it ranked in the top quartile of submissions.

There are two caveats to point out regarding our incarnation of the crowd prediction method for forecasting flu. First, the ILINet data we showed participants, and also asked them to predict, was subject to weekly revision—in some cases significantly so (for example, wILI in HHS region 2 on 2015w03 was first reported as 6.2%, and then the next week as 5.6%; the final, stable value was reported as 5.0%). The changing values are due to a backfill process whereby data from late-reporting providers is used to retrospectively update prior values of wILI. This is one reason, for example, that MAE *after* the Peak Week is non-zero; even once the peak has been observed with high confidence, there is still some non-negligible chance that a subsequent update due to backfill will result in a revision of the peak timing. A further discussion of the effects of backfill can be found in S1 Text. Second, the data used in our present analysis is the same data collected for the various contests, but our methodology in the 2014–2015 flu contest differed slightly from what we present here. Namely, our 2014–2015 contest submissions assumed a normal distribution over user inputs, whereas here, and in our 2015–2016 contest submissions, we assumed a Student’s t distribution.

### Advantages and disadvantages

There are several important limitations of the human judgment approach relative to purely data-driven methods that should be made clear. First, these results are only representative of two flu seasons and a single chikungunya outbreak. This highlights one of the biggest shortcomings of this approach—collecting predictions is a tedious and time-consuming process. Unlike statistical methods which can be applied retrospectively to any outbreak, the approach here requires a significant amount of work from a large number of participants. For example, because of this we are unable to perform cross validation across seasons. Second, these results do not necessarily provide us with an improved understanding of epidemiological dynamics. In contrast, statistical methods aim to learn from past data in order to better describe and model the epidemic process. On the other hand, the human judgment approach does have unique advantages over purely data-driven systems. Humans have the innate and powerful ability to assimilate, with little to no effort, diverse data sources and considerations. An example of this is using news headlines, which we display within the Epicast interface, to inform predictions. Another advantage of human judgment is the ability to make reasonable predictions for events with little historical precedent, like the outbreak of a new disease or a disease invasion in a new location.

The task of predicting trajectories is not trivial, and we asked each of the participants to provide us with many such trajectories over quite a long period of time. This resulted in some tedium, which we suspect is the reason for the relatively high attrition rate in the flu Epicast. There are many guidelines describing ways to make crowd work streamlined and sustainable, and we made every effort to implement these ideas. To minimize the overall amount of effort required and to streamline the process as much as possible we: allowed participants to use their previously entered forecasts as a starting point; accepted any number of regional predictions (not requiring all eleven to be completed); reduced the entire process to one drag and one click per region; and sent URLs tailored with a unique identifier via email each week to bypass having to manually login. Additionally, we tried to increase interest and participation by including a leader board of both weekly and overall high scores. We also had the competing objective of collecting the most informed forecasts from the participants. To this end we included a section of links to educational resources, and for the flu Epicast we embedded within each participant’s home page a Google news feed on the topic of “flu”.

Epicast is not well suited for all forecasting situations. “Wisdom of crowds” methods are robust to high variance among individual predictions, but require that the overall distribution of predictions is unbiased. By showing wILI trajectories of past flu epidemics on Epicast’s forecasting interface, we undoubtedly bias predictions toward typical flu seasons. While this may be beneficial when forecasting a typical flu season, it is almost certainly harmful when predicting highly atypical flu epidemics, and especially pandemics. Epicast is not robust to “long-tail” events such as these. In general, Epicast is best suited for situations where: (1) the event is regularly occurring, (2) historical surveillance data is available for many examples of the event, and (3) ongoing surveillance data is available with relatively short lag in comparison to the length of the event. This may explain the increased difficulty of Epicast in predicting the chikungunya invasion: it was a one-time event with no historical data and relatively lagged and intermittently available ongoing data.

Alternative prediction methods which solicit and aggregate human judgment exist; the Delphi method [38] is one such example. Both the Delphi method and the Epicast method herein collect predictions from human participants and produce a single output prediction. However, the Delphi method is iterative, requiring more time, effort, and coordination. The Delphi method requires from each participant not only a prediction, but also reasoning or justification for that prediction. Then, all participants are shown the predictions, and reasons for them, of all other participants. Participants are then given the opportunity to revise their predictions, and the process continues iteratively either until convergence or some other stopping criteria are met. One of the design goals of Epicast was to minimize human time and effort required, and so we did not pursue the Delphi method. However, it would be of value to compare the Epicast and Delphi methods to learn the relative advantages and disadvantages of each method in terms of both human effort and prediction accuracy.

### Future work

It was our hope that the number of participants would grow organically, for example through word of mouth and social media. Instead, we found it difficult to recruit new participants and to maintain participation throughout the flu season. The failure to achieve a true “crowd” is most likely due to the tedium of the task, and we are working on ways to both reduce this tedium and to make the task more gratifying for participants. While we strove to design the user interface in a way that minimizes the level of effort required to input predictions, there is always room for further improvement. One option we considered, but did not implement because of the small number of participants, is to reduce workload by asking participants to provide a prediction only for a randomized subset of regions. Another option we considered, but did not completely implement due to time constraints, was gamification. This was partially implemented in the form of leader boards, but it would be difficult to provide a more immediate reward because of the inherent delay between prediction and revelation of true outcomes.

There are several additional ways in which the Epicast method could be improved. First, there is an important relationship between a prediction, and the level of confidence in that prediction, that we were unable to capture. We asked participants to give us their best point predictions, but there was no way for them to communicate with us their level of confidence in those and other predictions—a forecast. We made the implicit assumption that disagreement among user predictions implies lack of confidence, which is probably true to some extent. The inverse however—that uniformity in predictions implies high confidence—is clearly untrue. Consider as an example the case where everyone believes that next week’s wILI has a 60% chance of staying the same as this week’s wILI, resulting in all point predictions strongly concentrated on the same wILI, and the distributional spread being very narrow, in contrast with the participants’ beliefs. It would be ideal to collect from each user a more informative measure of their confidence, but this would undoubtedly complicate the user interface and degrade the overall experience (which we were averse to).

Another improvement to consider is a weighted combination of predictions whereby participants who have historically had more accurate predictions are given more weight in the aggregation process. This is similar in spirit to weighting user recommendations and rankings, which has been shown to increase accuracy in those settings [39, 40]. In the case of Epicast, there is limited evidence suggesting that some participants are overall more (or less) accurate than other participants. One example of this is in Fig 5B where one participant has significantly higher Win Rate than Epicast and several other users. On the other hand, it is not clear whether the variance of prediction error is sufficiently small to learn which users are the most accurate in a reasonable amount of time—before the epidemic peak, for example. In other words, differences in *accuracy* may be exploitable, but only if *precision* is sufficiently high. If this is the case, then an adaptive weighting scheme could benefit the overall forecast. However, there is a critical obstacle that hinders the practical implementation of such a scheme: because of backfill, the final measure of accuracy is not known for many months. Despite this, we propose, implement, and analyze one such scheme in S1 Text—the result is a small and statistically insignificant increase in accuracy.

A natural evolution of systems such as those for epidemiological forecasting is the combination of human and statistical (machine) methods [41, 42]. The first question in such a project is whether human predictions should be given as input to statistical methods or whether the output of the statistical methods should be shown to humans for more informed predictions. In theory both directions are viable, and there are intuitive reasons for each. In support of the latter, people are naturally inclined to trust forecasts made by humans (or to distrust forecasts made by machines), a phenomenon known as algorithm aversion [43]. Supporting the former, on the other hand, is the observation that in many settings and in a variety of tasks, objective machine prediction is often superior to subjective human prediction [44, 45]. We have begun to explore both directions; currently, we show a subset of Epicast participants a confidence band derived from a separate statistical method, and we are developing a retrospective analysis wherein we compare performance of various statistical methods with and without a supplemental input of human prediction as an independent data source. In the meantime, we plan to continue to host Epicast and collect predictions for the current flu season, and we end this section with an open invitation to participate [34].

### Conclusion

For years, both humans and machines have been employed to tackle difficult prediction problems, and the biases involved and the relative advantage of data-driven approaches are at least well documented [43, 44], if not well understood. We do not make the claim that human judgment is intrinsically more valuable or more capable than machines when making epidemiological forecasts, but we do posit that there is value in understanding the strengths in each approach and suspect that both can be combined to create a forecasting framework superior to either approach alone.

## Supporting information

S1 Text Supporting Information. This document includes additional results and analysis, including a breakdown by each HHS Region, forecasts of week of epidemic onset, performance of a subset of “expert” users, and an adaptive weighting scheme.
(PDF)

S1 Data Forecasts and ground truth. (ZIP)
