# Supplementary material for: A human judgment approach to epidemiological forecasting
Source: PLoS Comput Biol. 2017 Mar 10;13(3):e1005248. doi: 10.1371/journal.pcbi.1005248 (PMC5345757; doi:10.1371/journal.pcbi.1005248)
Supplement: S1 Text — This document includes additional results and analysis, including a breakdown by each HHS Region, forecasts of week of epidemic onset, performance of a subset of “expert” users, and an adaptive weighting scheme. (PDF) [file pcbi.1005248.s001.pdf]

A Human Judgment Approach to Epidemiological Forecasting

David C. Farrow Logan C. Brooks Sangwon Hyun Ryan J. Tibshirani
Donald S. Burke Roni Rosenfeld

Supporting Information

Contents

1 Breakdown by HHS Region **1**
1.1 Region 1 . . . 2
1.2 Region 2 . . . 3
1.3 Region 3 . . . 4
1.4 Region 4 . . . 5
1.5 Region 5 . . . 6
1.6 Region 6 . . . 7
1.7 Region 7 . . . 8
1.8 Region 8 . . . 9
1.9 Region 9 . . . 10
1.10 Region 10 . . . 11
1.11 National . . . 12

2 Analysis of 2014–2015 Onset Week **13**
2.1 Accuracy on Forecasts of Onset . . . 13
2.2 Backfill Effects . . . 14

3 Expert versus Non-expert Forecasts **16**
3.1 Comparison of MAE and MLL . . . 16
3.2 Win Rate Analysis . . . 17

4 An Adaptive Weighting Scheme **19**
4.1 Strategy . . . 19
4.2 Results . . . 19

1 Breakdown by HHS Region

In all main text analysis, Epicast (influenza) error was averaged over the eleven Health and Human Services (HHS) regions [1] (including the "National" super-region). Here we break down the analysis by region, showing that some regions are more difficult to predict than others. The reasons for this are varied, but include at least differences in population, reporting, and climate. Still, the general trend holds; Epicast is usually more accurate than individual users and automated forecasts, especially for short-term targets.

In what follows, the top panel shows a map of the states within the region, the middle panel plots mean absolute error (MAE) of Epicast as a function of lead time relative to the Peak Week (as in main text Fig. 6), and the bottom panel compares Win Rate of Epicast against individual predictions (as in main text Fig. 5). Lead time refers to the number of weeks spanning the week on which the forecast was made to the week on which wILI reaches its peak value (positive before the peak, negative afterwards). Win Rate refers to the fraction of all predictions in which Epicast has lower absolute error than another forecaster. The statistical significance of the Win Rate is determined by Sign test at $p < 10^{-2}$ (*) and $p < 10^{-5}$ (**).

1.1 Region 1: *CT, MA, ME, NH, RI, VT*

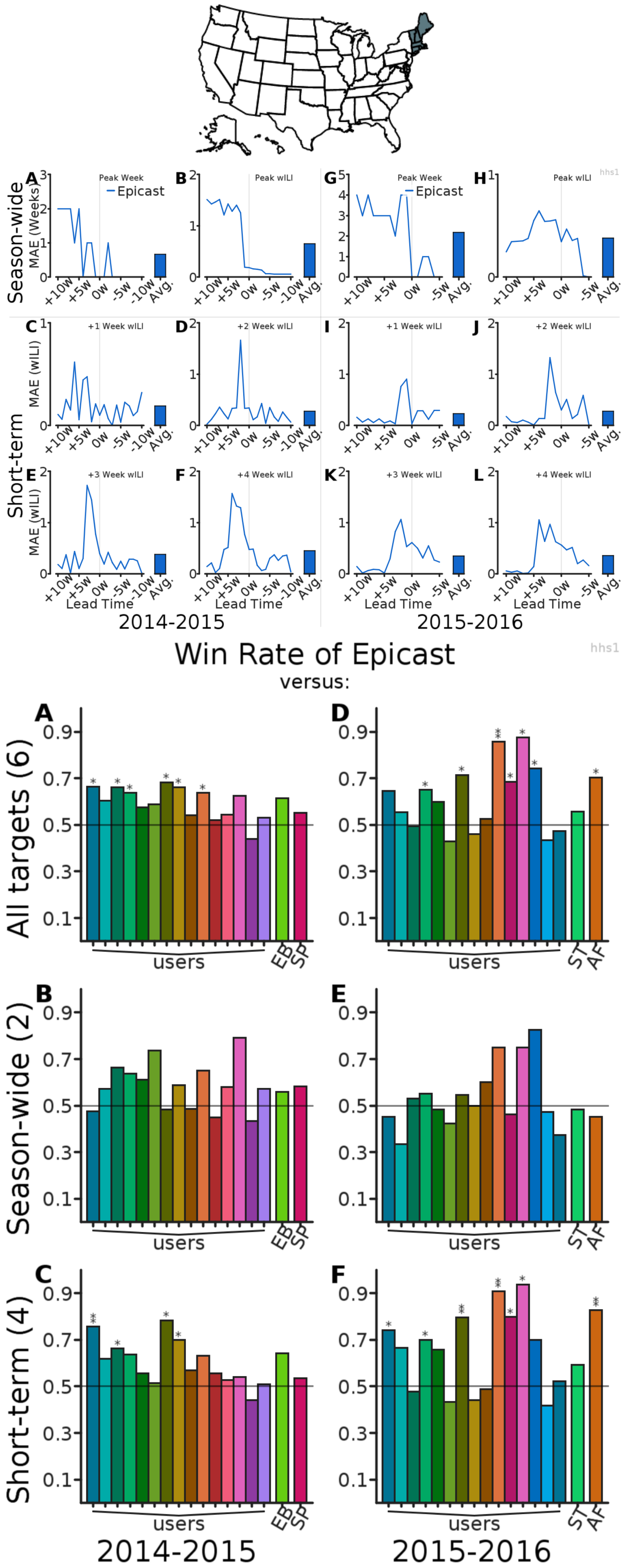

Figure 1. Region 1 Map, MAE, and Win Rate.

1.2 Region 2: *NJ, NY*

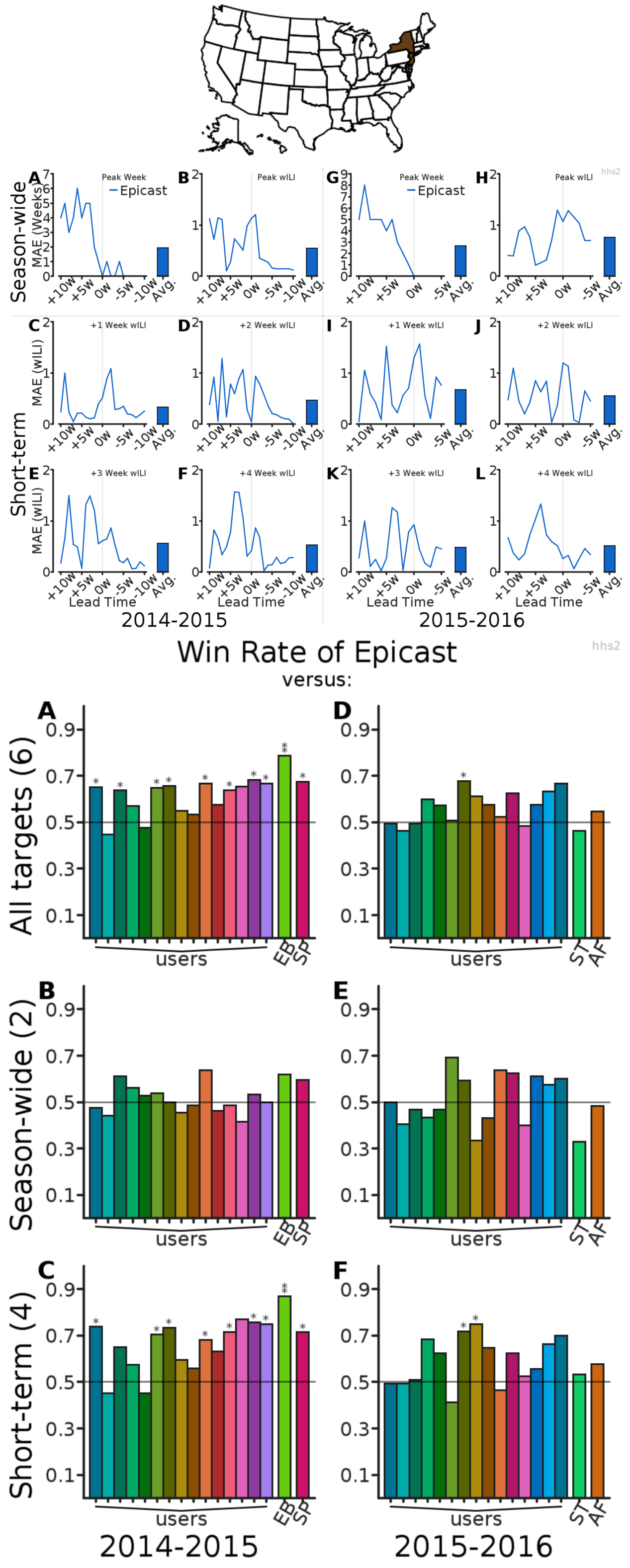

Figure 2. Region 2 Map, MAE, and Win Rate.

1.3 Region 3: *DE, MD, PA, VA, WV*

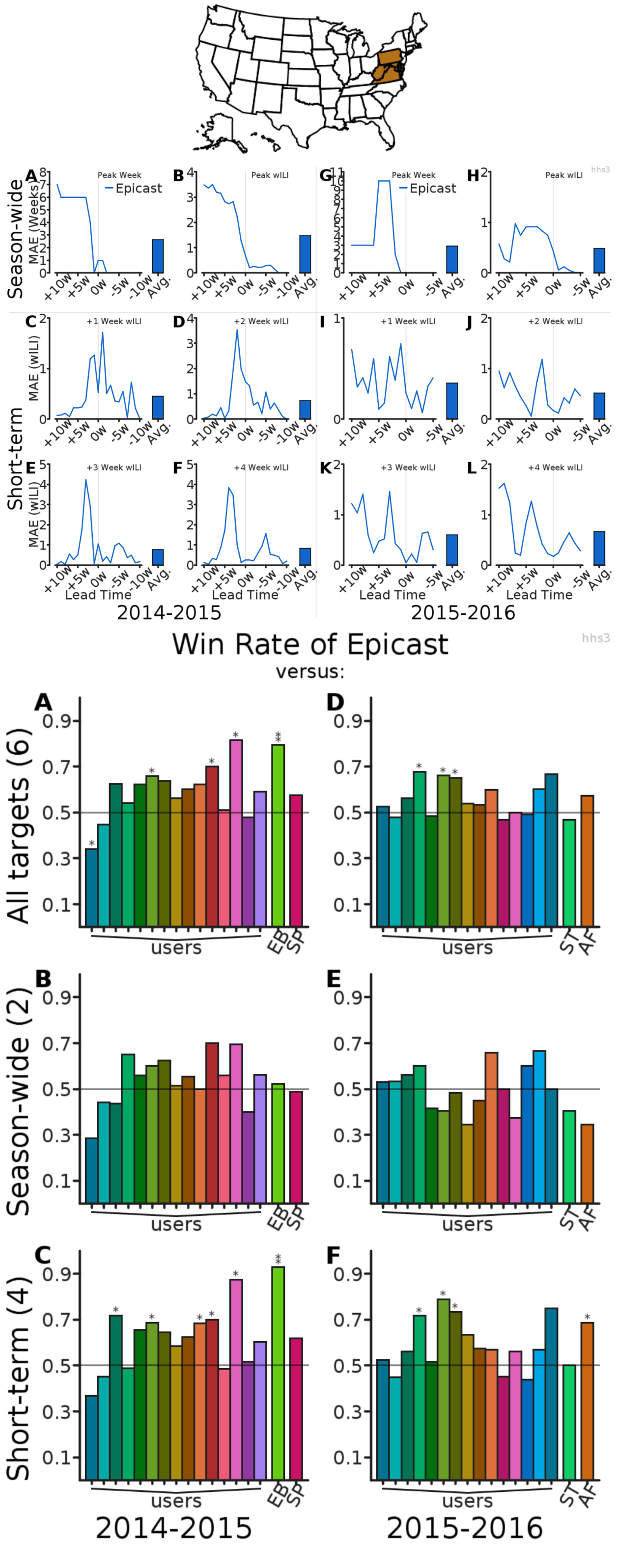

Figure 3. Region 3 Map, MAE, and Win Rate.

1.4 Region 4: *AL, FL, GA, KY, MS, NC, SC, TN*

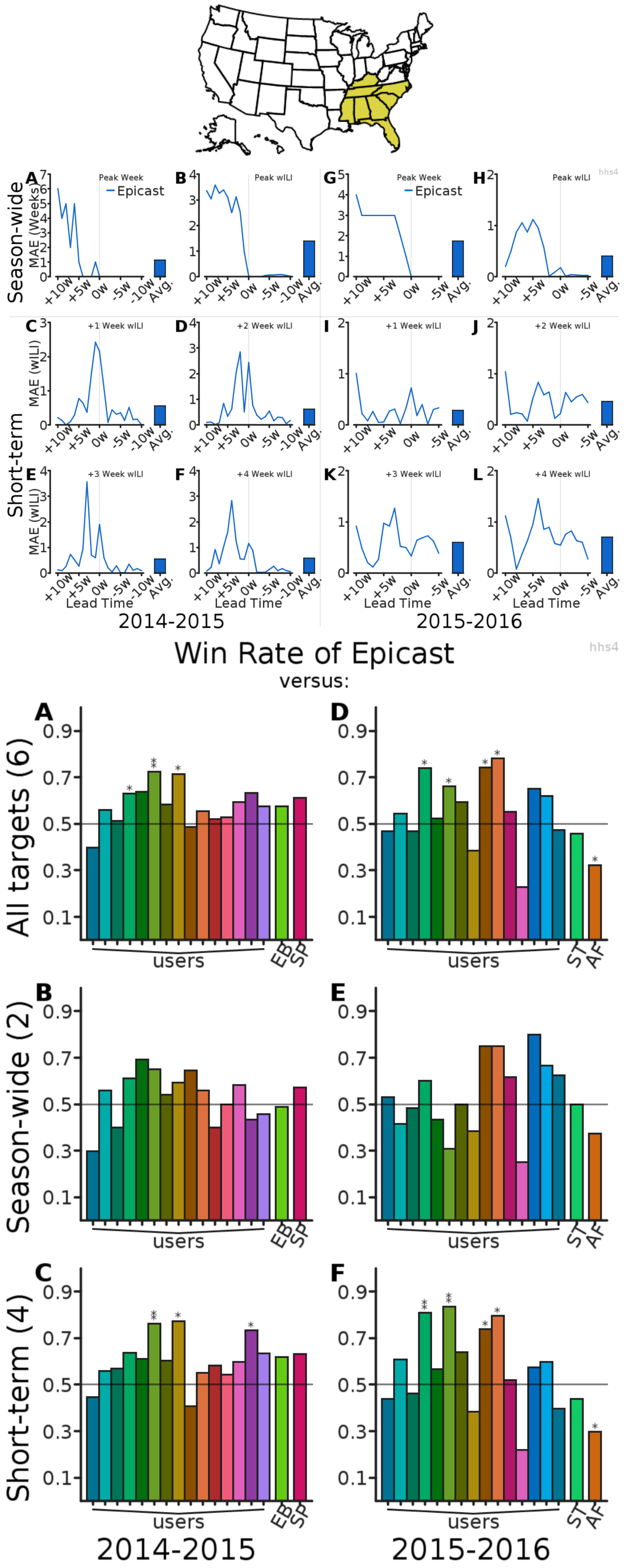

Figure 4. Region 4 Map, MAE, and Win Rate.

1.5 Region 5: *IL, IN, MI, MN, OH, WI*

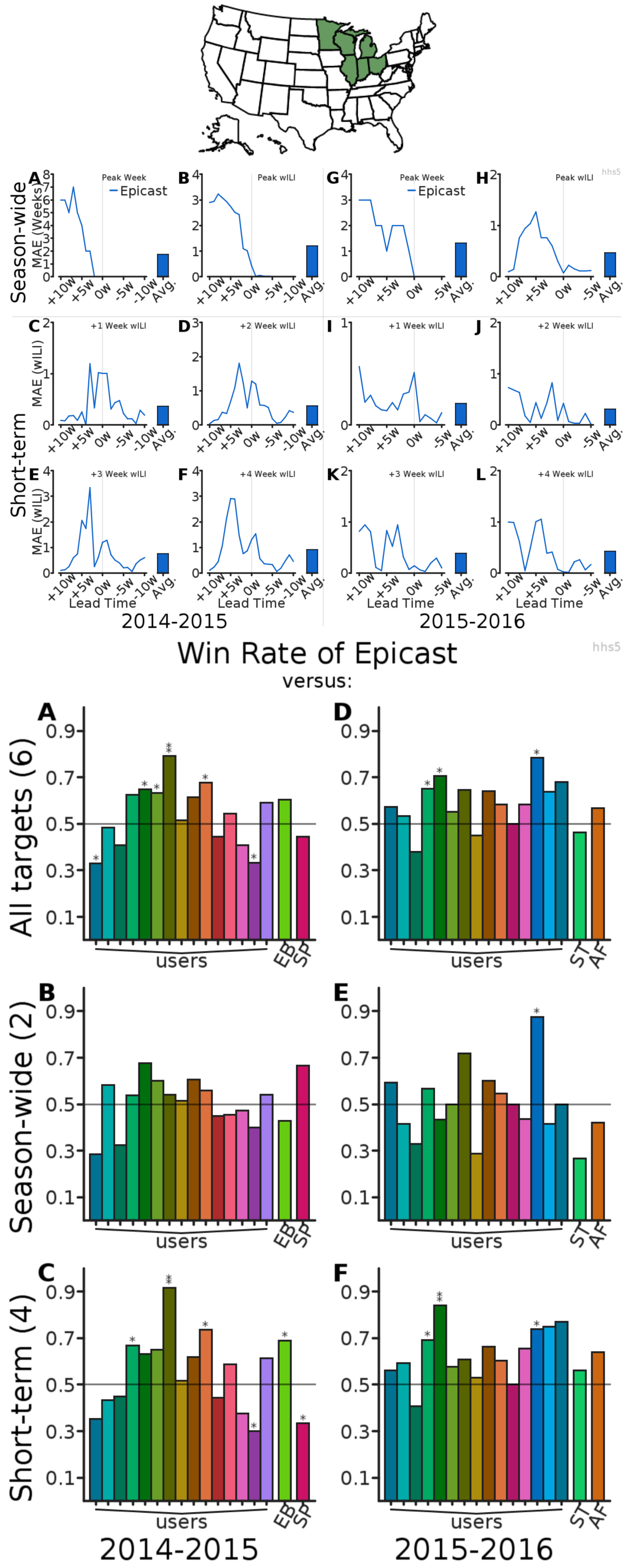

Figure 5. Region 5 Map, MAE, and Win Rate.

1.6 Region 6: *AR, LA, NM, OK, TX*

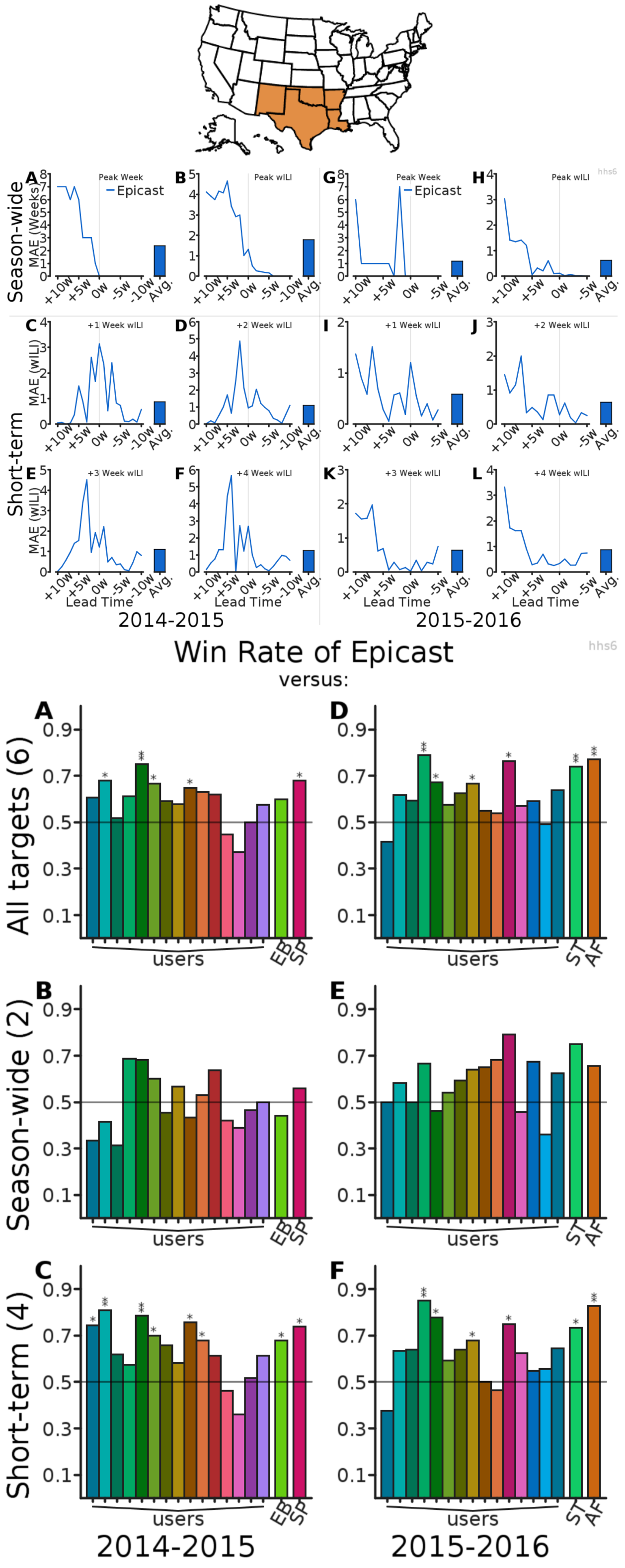

Figure 6. Region 6 Map, MAE, and Win Rate.

1.7 Region 7: *IA, KS, MO, NE*

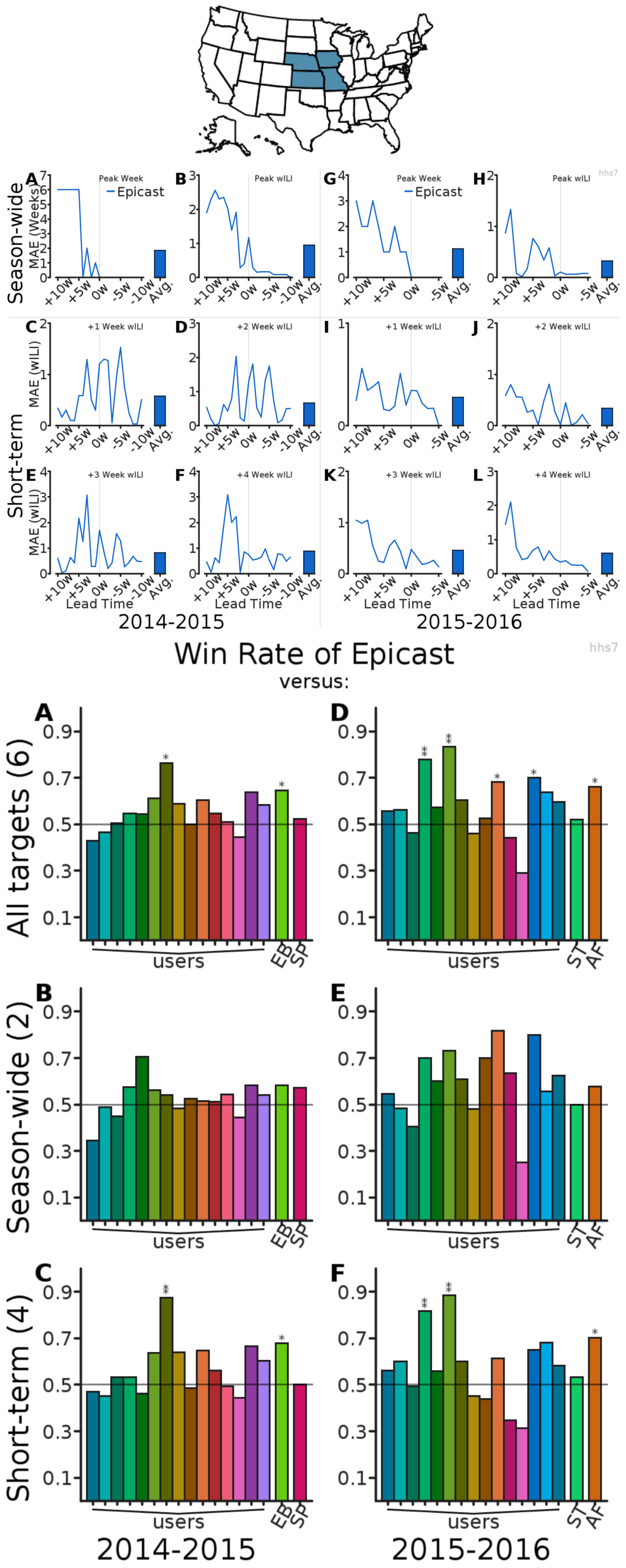

Figure 7. Region 7 Map, MAE, and Win Rate.

1.8 Region 8: *CO, MT, ND, SD, UT, WY*

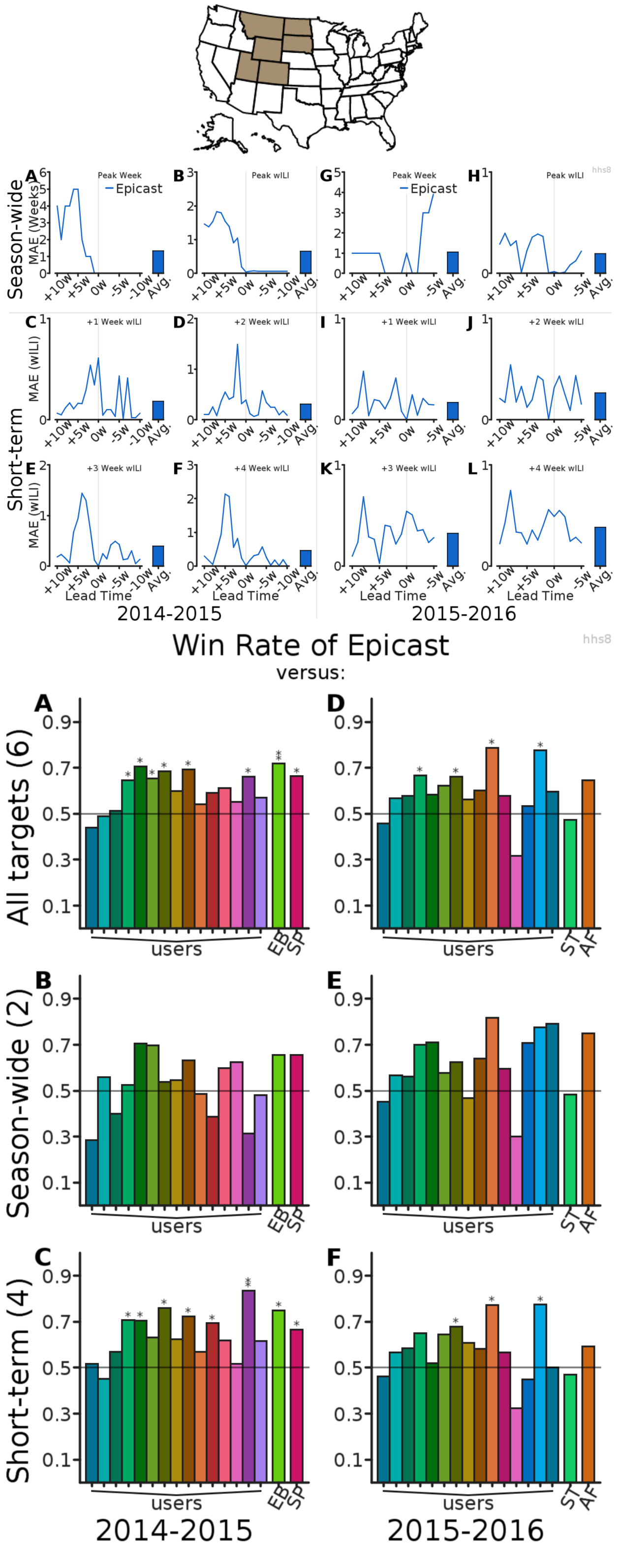

Figure 8. Region 8 Map, MAE, and Win Rate.

1.9 Region 9: *AZ, CA, HI, NV*

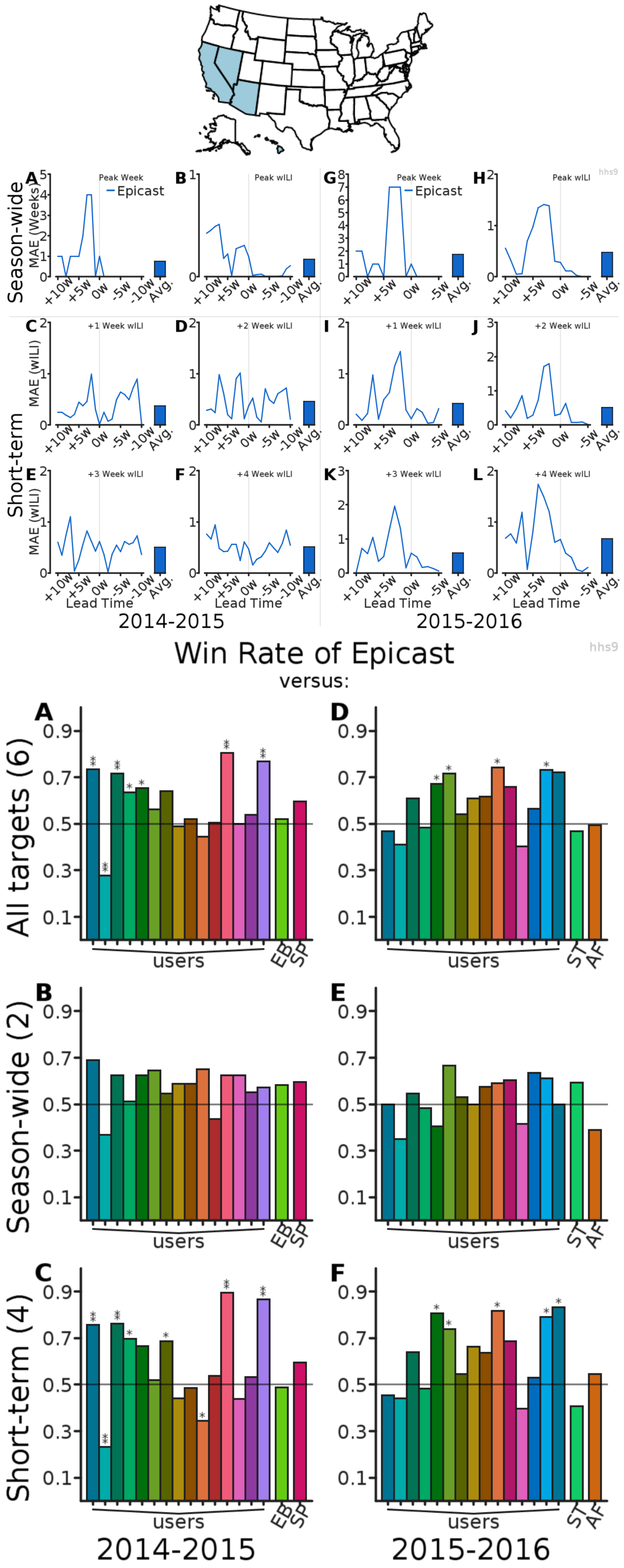

Figure 9. Region 9 Map, MAE, and Win Rate.

1.10 Region 10: *AK, ID, OR, WA*

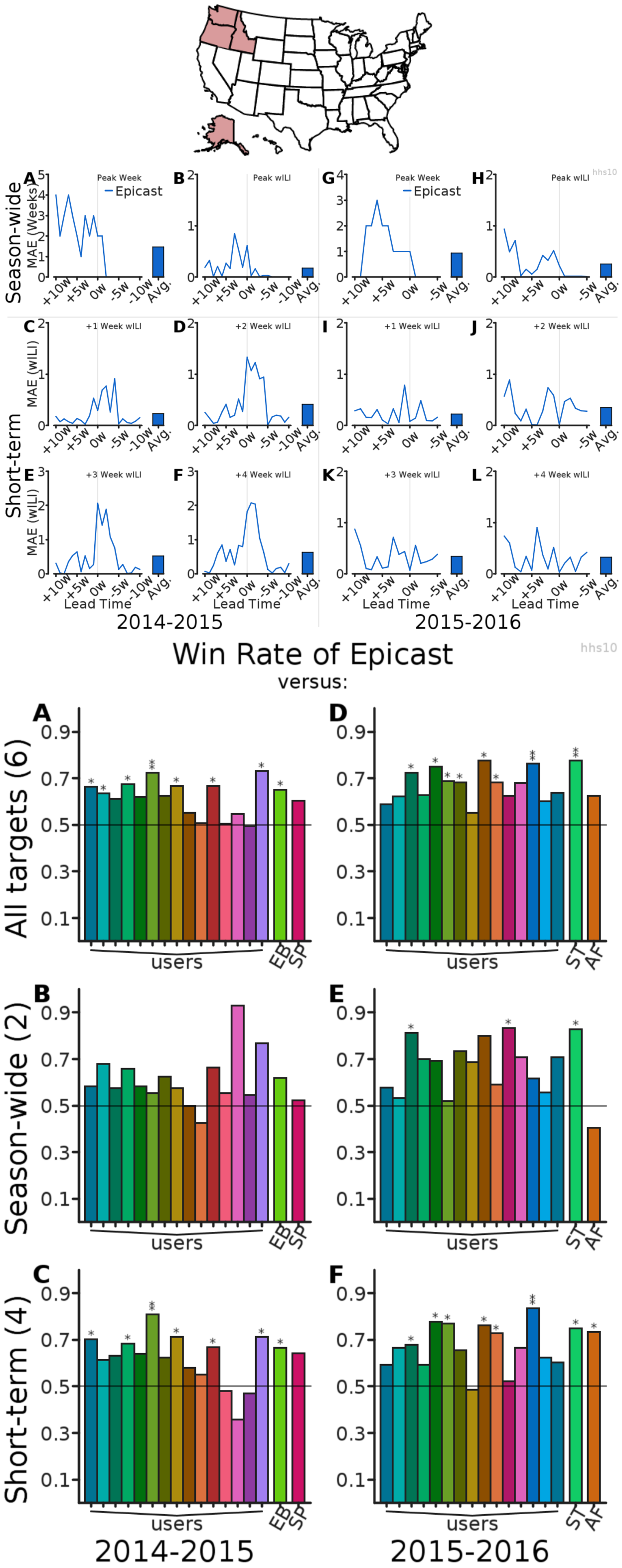

Figure 10. Region 10 Map, MAE, and Win Rate.

1.11 National: *All States, D.C., and Territories*

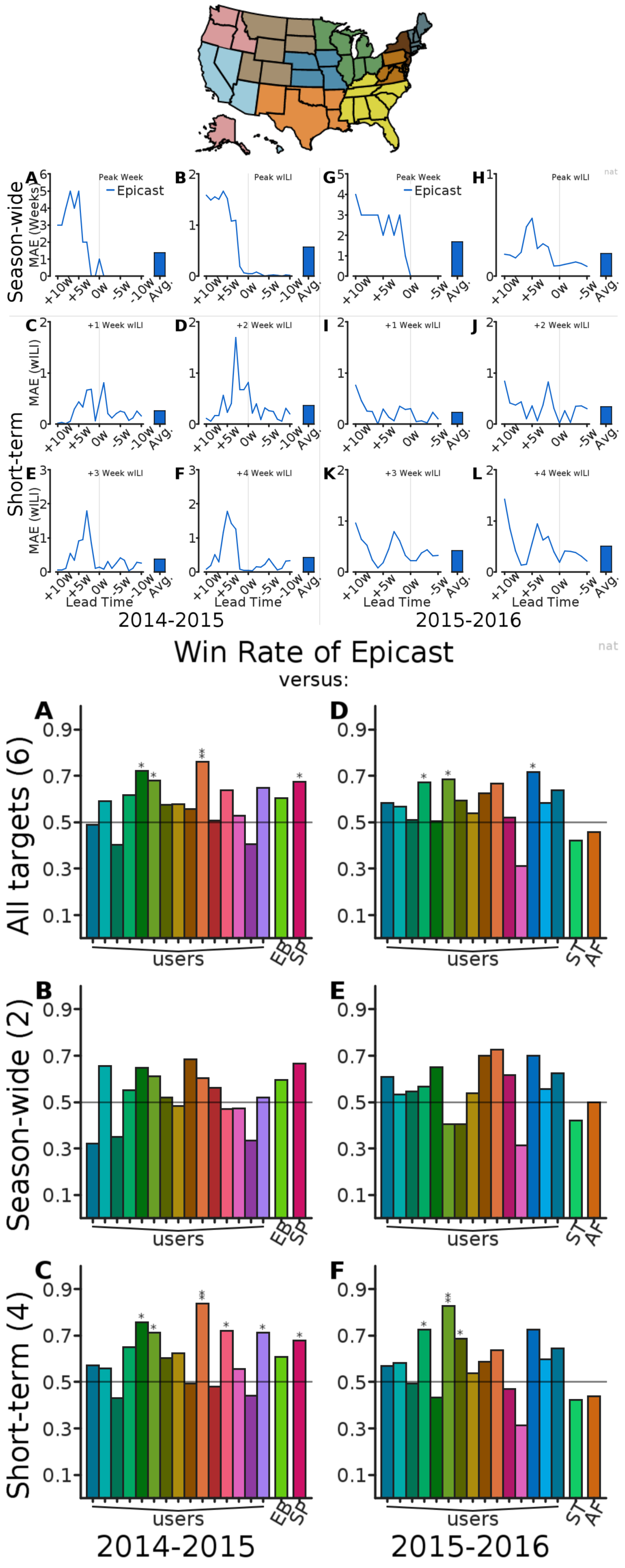

Figure 11. National Map, MAE, and Win Rate.

2 Analysis of 2014–2015 Onset Week

In addition to the six influenza targets described in the main text, CDC required one additional season-wide target for contest submissions: the week on which the epidemic onset. Onset week is defined as the first week on which wILI—*rounded to 1 decimal place*—is at or above the region-specific baseline, for at least three consecutive weeks. These regional baselines are updated annually; for the 2014–2015 flu season they were 1.2, 2.3, 2.0, 1.9, 1.7, 3.3, 1.7, 1.3, 2.7, 1.1, and 2.0 for HHS Regions 1-10 and National, respectively [2].

2.1 Accuracy on Forecasts of Onset

Here we repeat the standalone and comparative analyses of Epicast performance in forecasting onset week (Fig. 12). Due to an early epidemic onset in most regions, our analysis by lead time is limited to a window of 6 weeks. As before, we find that when considering pairwise ranking in absolute error, no individual user or system has a statistically significant Win Rate over Epicast. Two users do win over half the time, but the result doesn't reach the $p < 10^{-2}$ threshold of significance. On the other hand, Epicast beats the two statical systems—one significantly. As with the other season-wide targets, we find that error in onset falls during the weeks preceding the onset week and then levels off. Unlike the other targets, error in onset week, by all measures, never reaches zero; predicted onset is always off by more than 1 week in at least one region, as is particularly evident in the plot of average log score. The reason for this apparent shortcoming is, as discussed in the next section and in the main text, due to backfill.

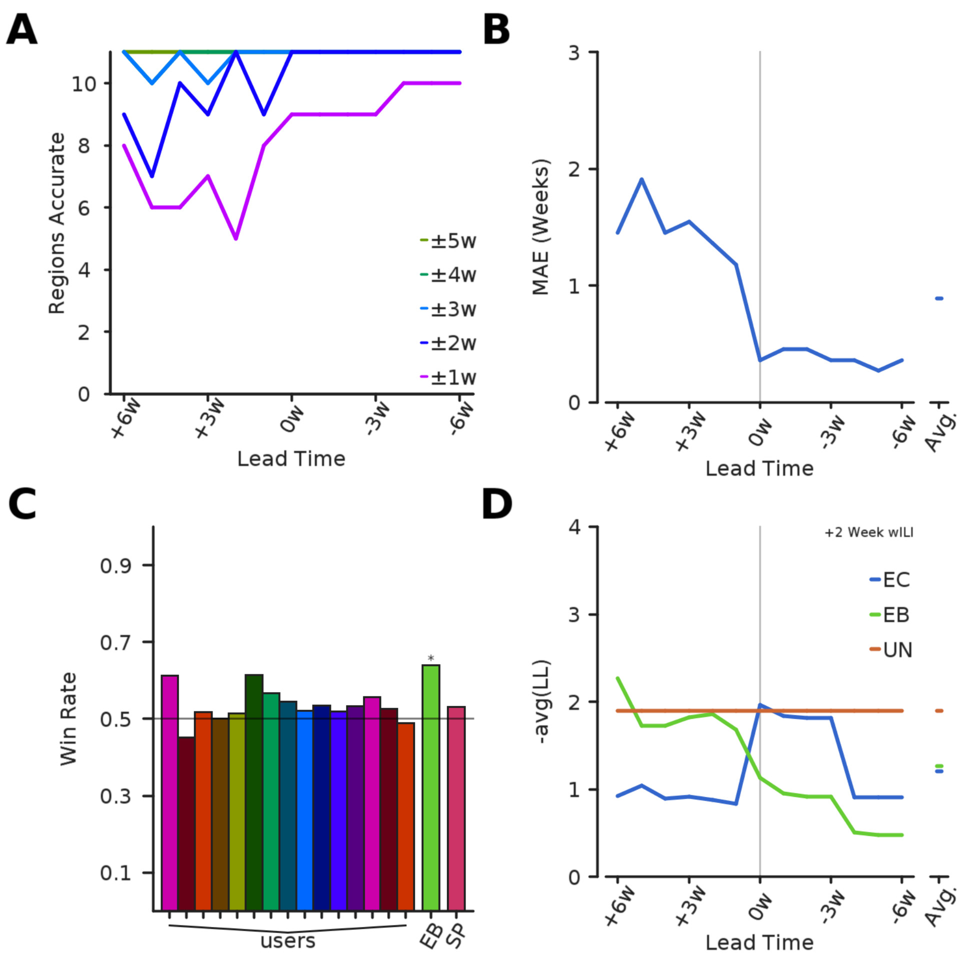

Figure 12. Onset Week. Epicast performance on an additional season-wide target, the week of epidemic onset. **(A)** As in main text Fig. 4, the number of regions in which Epicast's prediction falls within a given range of the actual onset week, plotted as a function of lead time relative to epidemic onset. **(B)** As in main text Fig. 6, MAE (across regions) in onset is plotted as a function of lead time relative to epidemic onset. **(C)** As in main text Fig. 5, pairwise Win Rate is shown for Epicast against individual users and statistical systems. **(D)** As in main text Fig. 7, average log score is shown for Epicast (EC), Empirical Bayes (EB), and the Uniform System (UN) as a function of lead time relative to epidemic onset.

2.2 Backfill Effects

wILI targets are robust to some changes in reported values; a forecast of 2.0 wILI is not so far off from a forecast of 2.1 wILI. By contrast, the target of onset week (and perhaps to a smaller extent, Peak Week) is fragile; small updates to published wILI values can have a large impact on the target week. For example, consider the scenario in HHS Region 1 (baseline = 1.2). On 2015w16 (20 weeks after actual onset), onset week measured on the most up-to-date data was 2014w48. One week later, an adjustment due to backfill caused wILI on 2014w49 to fall below the baseline, resulting in a new onset week of 2014w50. This small and delayed wILI revision, from 1.27 to 1.10, caused a two week shift in onset week (Fig. 13). A similar situation happened in HHS Region 2. To be clear, we neither attribute fault to nor place blame on any portion of the collection and reporting process; we simply point out one caveat of measuring weekly targets on this dataset.

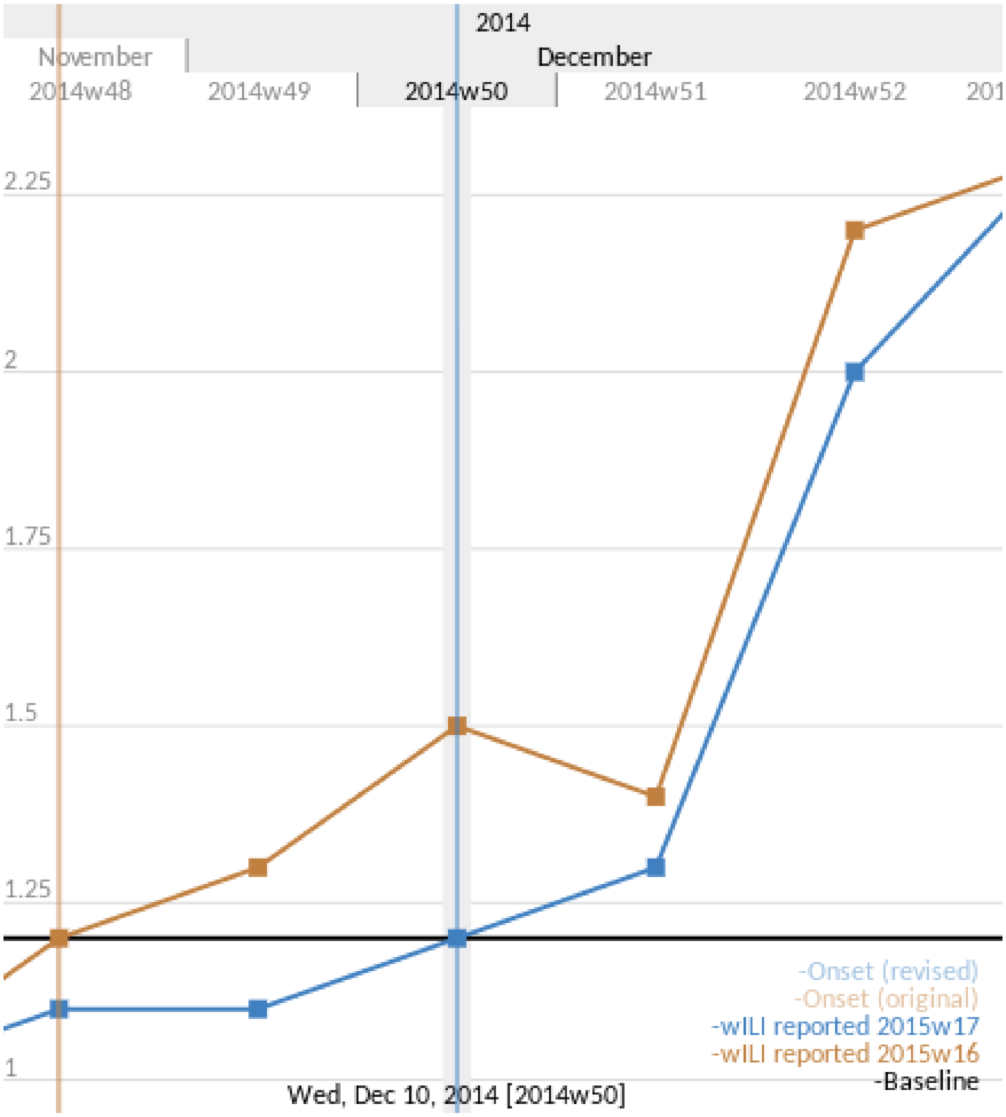

Figure 13. HHS1 Backfill. A large change (2 weeks) in onset week was caused by a small change (-0.17) in wILI.

3 Expert versus Non-expert Forecasts

An interesting question that arises in the context of wisdom of crowds forecasting is whether "experts" (by some definition) make better forecasts than non-experts. Within the online Epicast user interface, we gave users the option to self-classify themselves as having background or expertise in up to five areas: epidemiology, statistics and/or machine learning, virology, public health, and influenza. For the 2014–2015 season, of the 48 active users, 25 claimed expertise in at least one area, and the other 23 did not claim expertise in any area.

3.1 Comparison of MAE and MLL

To assess the relative performance of the "experts" versus the "non-experts", we build forecasts using only predictions made from each group, and we show MAE (Fig. 14) and MLL (Fig. 15) of each group as a function of lead time for each target. Perhaps unsurprisingly, the expert group generally has MAE and MLL less than or equal to that of the unmodified Epicast forecast and the Epicast based only on non-expert inputs. The most striking difference between the two groups appears when forecasting Peak Week, especially at a long lead time. On the other hand, accuracy between the two groups is essentially indistinguishable for the 1- and 2- Week Lookahead targets. The difference when predicting Peak Height and wILI at 3- and 4- Week Lookaheads is small, but noticeable.

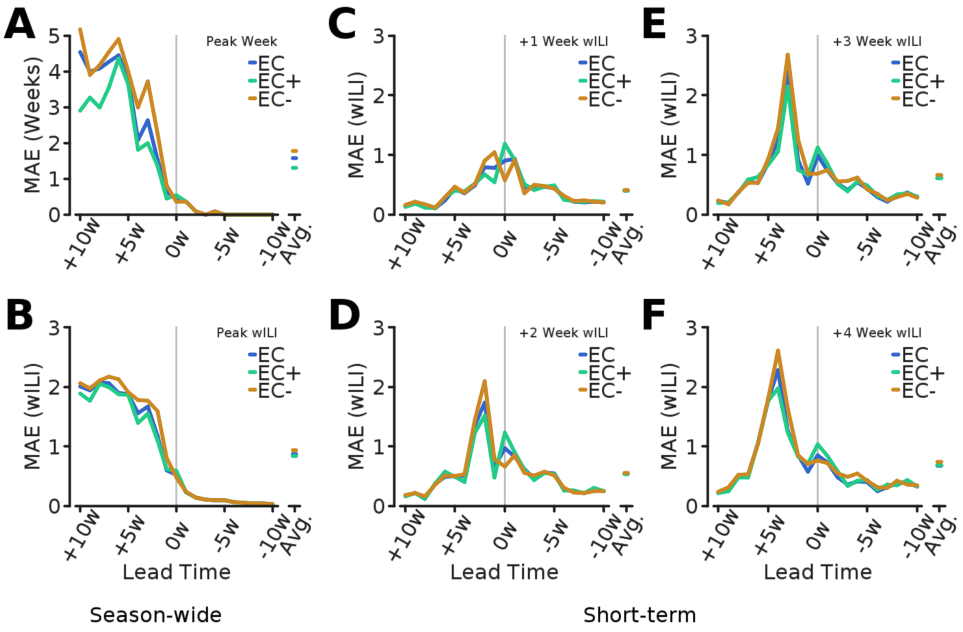

Figure 14. Expert versus Non-expert MAE. MAE is plotted for unmodified (all users) Epicast (EC), expert-based Epicast (EC+), and non-expert-based Epicast (EC-) as a function of lead time relative to the Peak Week. As in main text Fig. 6, panels **A, B, C, D, E, and F** show MAE separately for each target.

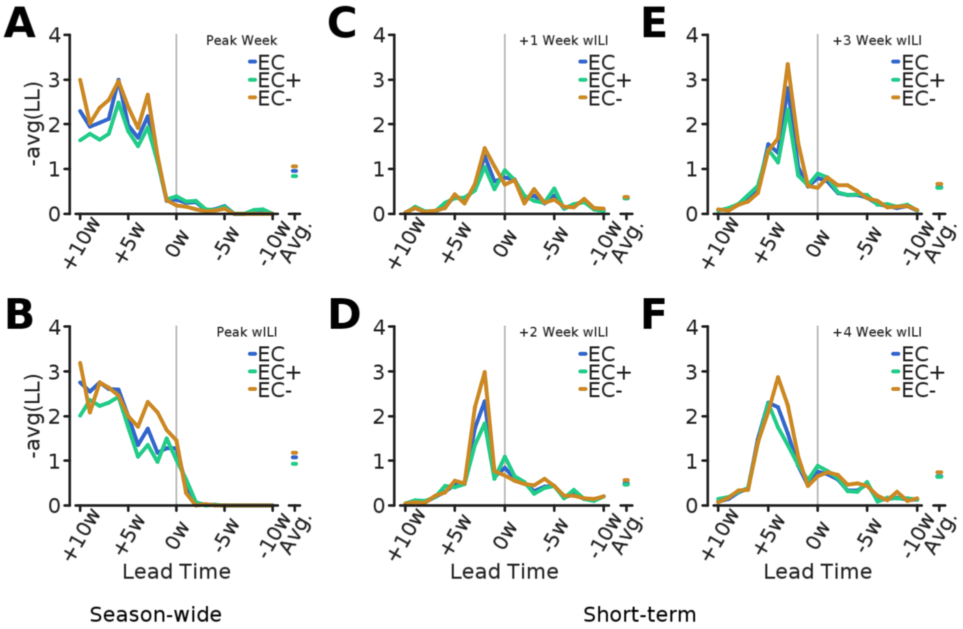

Figure 15. Expert versus Non-expert MLL. Mean log-likelihood is plotted for unmodified (all users) Epicast (EC), expert-based Epicast (EC+), and non-expert-based Epicast (EC-) as a function of lead time relative to the Peak Week. As in main text Fig. 7, panels **A, B, C, D, E, and F** show log score separately for each target.

3.2 Win Rate Analysis

To determine whether these observations are statistically significant, we use a Sign test as before, this time separately for each target (Fig. 16). Neither expert nor non-expert versions of Epicast have a significantly higher Win Rate than the Epicast built from all users. On other hand, none of the Win Rates reaches the $p < 10^{-2}$ threshold of significance. It is, however, clear that the expert group loses to unmodified Epicast less frequently than the non-expert group. This raises the question of whether some subset of—or a special weighting of—users could significantly improve Epicast performance. We consider this question in the next section.

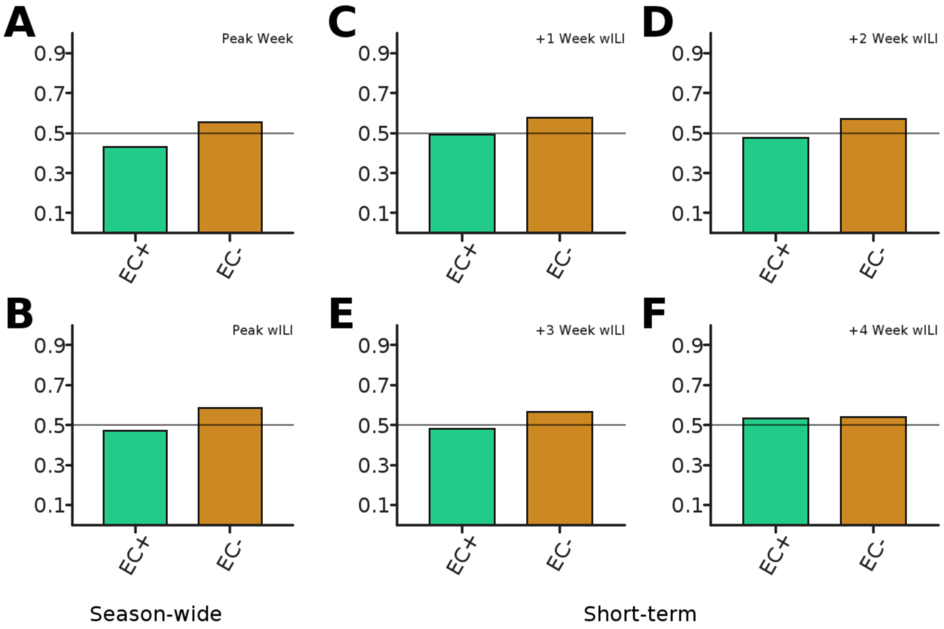

Figure 16. Win Rate of Epicast (all users) against Epicast (experts) and Epicast (non-experts). Win Rate of the unmodified (all users) Epicast system is plotted against expert-based Epicast (EC+) and non-expert-based Epicast (EC-). Epicast (all users) wins against non-experts more frequently than against experts. Panels **A, B, C, D, E, and F** show Win Rate separately for each target.

4 An Adaptive Weighting Scheme

Given that some users consistently outperform other users, is it possible to apply an adaptive weighting scheme to user predictions so that the overall accuracy of the Epicast is significantly improved? The primary obstacle to implementing such a scheme in *real-time* is that, due to backfill, it isn't possible to know which users are outperforming their peers. Still, we can estimate relative user performance using preliminary data and boost the weight of (hypothesized) high-accuracy users from week to week. We retrospectively attempt such a scheme for the 2014–2015 season below. Because we are limited in the number of user predictions, we can't exhaustively test adaptive weighting schemes. We think the one described here, though *ad-hoc*, is a reasonable approach.

4.1 Strategy

The original Epicast can be thought of as having a static weighting system wherein each user is given a weight of 1. Now we weight users based on two criteria: those who self-identify as experts, and those who had the lowest absolute error 2 weeks ago for a 3-week-ahead prediction of wILI (performance on the 3 Week Lookahead wILI target, after having seen the "truth" for two weeks). We select this particular target and timing for two reasons. First, it is difficult to differentiate user performance on very-short-term targets; predictions are very clustered at 1 week ahead and begin to spread out as the length of the prediction increases. At 3 weeks ahead, we first begin to notice that some users are outperforming other users. So then, why use the 3 Week Lookahead target and not the 4 Week Lookahead target? This leads to the second reason: we need data that is as stable as possible to determine who is performing well. Because of the previously discussed backfill issue, initial wILI estimates are unreliable. Reliability increases (backfill adjustments decrease) over time. The initial estimate is too unreliable to determine who is doing well, so we wait one additional week for the wILI value to settle a little bit closer to its final value. Finally, we have the additional constraint that we want to know as soon as possible who is doing well; we want to know as early as possible in the season who the accurate users are, and we want to be able to quickly adapt to changes in user performance. This method will allow us to rank users with a total lag of 5 weeks. To be more concrete, our weighting scheme is this: $w_u = 1 + e_u + r_u$, where w is the weight given to a prediction, e is 1 for (self-identified) experts (0 otherwise), and r is 1 for the top (arbitrarily) 5 users in terms of MAE on 3 Week Lookahead predictions (0 otherwise)—for each user u.

4.2 Results

To illustrate the adaptive nature of the weighting scheme proposed above, we show a moving average (over 5 weeks) of user weight as a function of submission week for the 5 users with the highest number of submissions (Fig. 17). As expected, some users are consistently given more weight than other users, and these weights change in response to accuracy of past predictions. To determine whether the Epicast built with these weights is better than the original Epicast, we show MAE (Fig. 18) and Win Rate (Fig. 19) for each target as before. Unsurprisingly, the weighted Epicast outperforms the original, unweighted Epicast. However, it is hard to say if there is any real advantage to using this particular weighting scheme, especially when considering the short-term targets. With the exception of the 4 Week Lookahead target, the weighted Epicast achieves a slightly higher Win Rate than the unweighted Epicast, but none of the Win Rates reach significance at the $p < 10^{-2}$ level. Still, these results suggest that there may be value in considering both user skill (self-classification as having expertise) and past performance (accuracy on previous predictions) when aggregating user predictions. We suspect that the advantage of a weighted Epicast would be much more meaningful with a larger, and more diverse, set of participants.

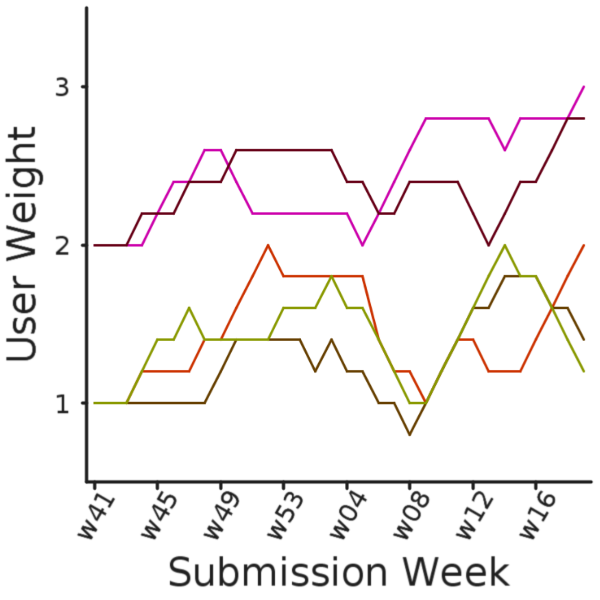

Figure 17. User Weight over Time. The five week moving average of user weight is shown for the 5 most active users.

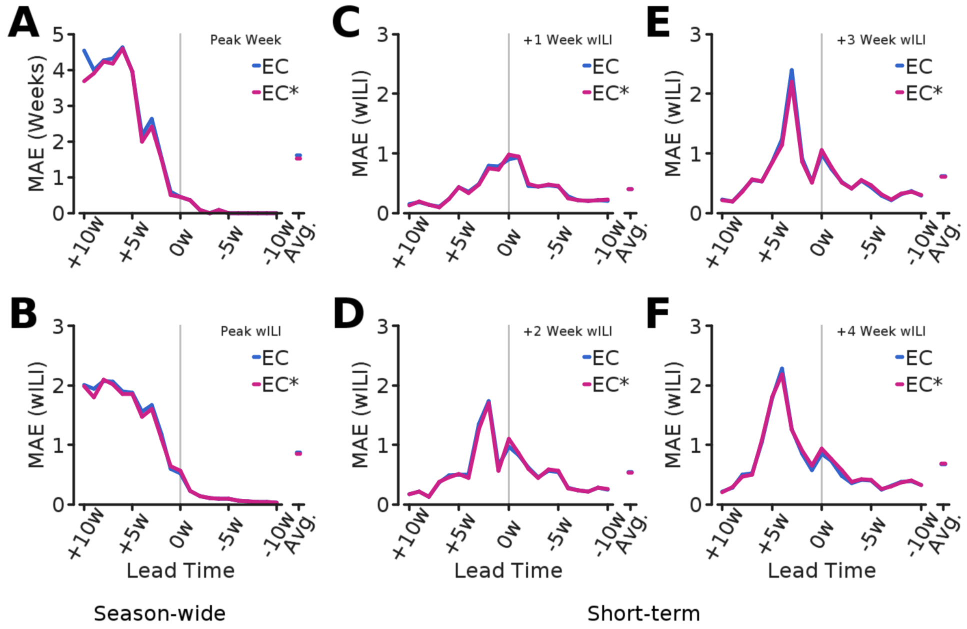

Figure 18. MAE of Weighted Epicast. MAE is plotted for unweighted Epicast (EC) and weighted Epicast (EC*) as a function of lead time relative to the Peak Week. As in main text Fig. 6, panels **A, B, C, D, E, and F** show MAE separately for each target.

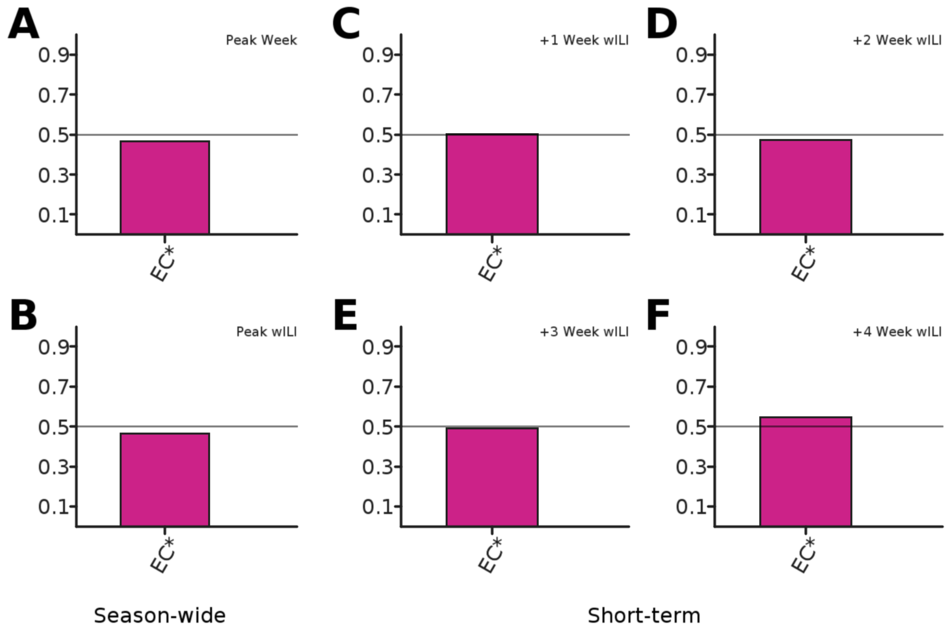

Figure 19. Win Rate against Adaptive Epicast. Win Rate of the unweighted Epicast system is shown against the weighted Epicast (EC*). Panels **A, B, C, D, E, and F** show Win Rate separately for each target.

References

[1] U.S. Department of Health & Human Services, *Regional Offices*, Available from: `http://www.hhs.gov/about/agencies/iea/regional-offices/index.html`.

[2] U.S. Centers for Disease Control and Prevention, *FluView Interactive*, Available from: `http://www.cdc.gov/flu/weekly/fluviewinteractive.htm`.
